# Advancing Lateral Flow Detection in CRISPR/Cas12a Systems Through Rational Understanding and Design Strategies of Reporter Interactions

**DOI:** 10.3390/bios15120812

**Published:** 2025-12-13

**Authors:** Irina V. Safenkova, Maria V. Kamionskaya, Dmitriy V. Sotnikov, Sergey F. Biketov, Anatoly V. Zherdev, Boris B. Dzantiev

**Affiliations:** 1 A.N. Bach Institute of Biochemistry, Research Centre of Biotechnology of the Russian Academy of Sciences, 119071 Moscow, Russia; mv.kamionskaya@fbras.ru (M.V.K.); sotnikov-d-i@mail.ru (D.V.S.); zherdev@inbi.ras.ru (A.V.Z.); dzantiev@inbi.ras.ru (B.B.D.); 2 State Research Center for Applied Microbiology & Biotechnology, 142279 Obolensk, Russia; biketov@mail.ru

**Keywords:** CRISPR/Cas12a, reporter, trans-target, lateral flow strip, point-of-care testing, *Erwinia amylovora*

## Abstract

CRISPR/Cas12a systems coupled with lateral flow tests (LFTs) are a promising route to rapid, instrument-free nucleic acid diagnostics due to conversion target recognition into a simple visual readout via cleavage of dual-labeled single-stranded DNA reporters. However, the conventional CRISPR/Cas12a–LFT system is constructed in a format where the intact reporter should block nanoparticle conjugate migration and can produce false-positive signals and shows strong dependence on component stoichiometry and kinetics. Here, we present the first combined experimental and theoretical analysis quantifying these limitations and defining practical solutions. The experimental evaluation included 480 variants of LFT configuration with reporters differing in the concentration of interacting components and the kinetic conditions of the interactions. The most influential factor leading to 100% false-positive results was insufficient interaction time between the components; pre-incubation of the conjugate with the reporter for 5 min eliminated these artifacts. Theoretical analysis of the LFT kinetics based on a mathematical model confirmed kinetic constraints at interaction times below a few minutes, which affect the detectable signal. Reporter concentration and conjugate architecture represented the second major factors: lowering reporter concentration to 20 nM and using smaller gold nanoparticles with multivalent fluorescent reporters markedly improved sensitivity. The difference in sensitivity between various LFT configurations exceeded 50-fold. The combination of identified strategies eliminated false-positive reactions and enabled the detection of up to 20 pM of DNA target (the *hisZ* gene of *Erwinia amylovora*, a bacterial phytopathogen). The strategies reported here are general and readily transferable to other DNA targets and CRISPR/Cas12a amplification-free diagnostics.

## 1. Introduction

Point-of-care testing (POCT) systems provide rapid, cost-effective, and easy-to-use diagnostic tools that enable on-site analysis without the need for complex instrumentation, providing timely results in both laboratory and field settings [1,2,3]. The demand for POCT capable of detecting viral and bacterial pathogens continues to grow across clinical, environmental, and food safety applications where rapid monitoring and control are essential [4]. Traditionally, immunoassay-based methods—particularly the lateral flow immunoassay (LFIA)—have been regarded as the gold standard in POCT because of their rapidity, simplicity, and high specificity [5]. One of the limitations of LFIAs is their comparatively low sensitivity, which is inferior to molecular diagnostic methods. To enhance assay sensitivity, various strategies have been employed, the most effective of which include modifying the detectable label (e.g., gold nanoparticles) [6] or replacing it with more efficient labels, such as fluorescent metal–organic frameworks [7], catalytically active nanozymes [8], or quantum dots [9].

**Figure 1:**
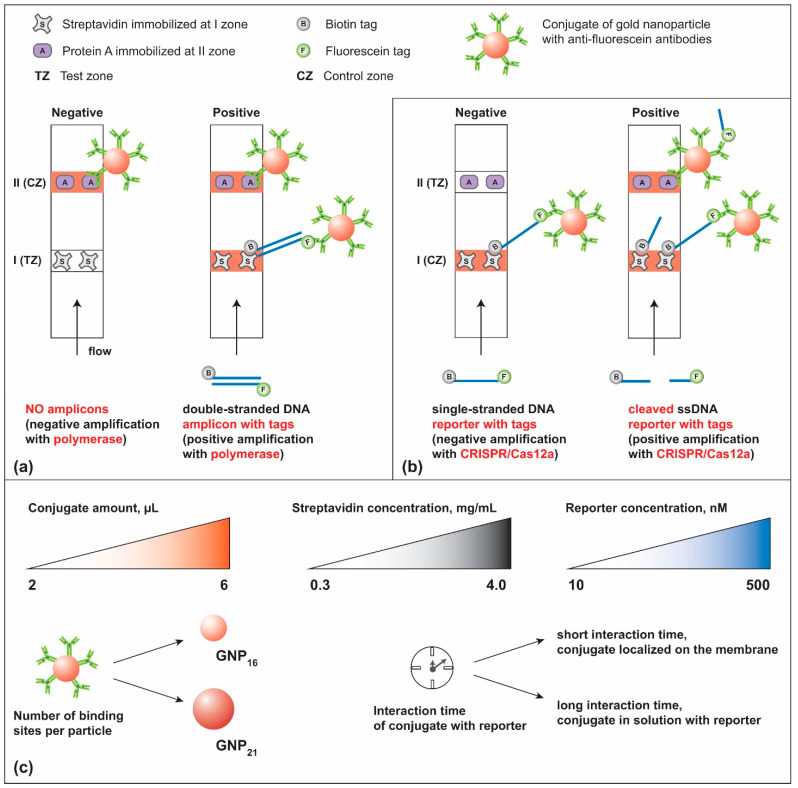
Scheme of NALFA strip for the detection of dsDNA amplicons with tags after isothermal amplification (**a**). Scheme of NALFA strip for detection of cleaved ssDNA reporters with tags after CRISPR/Cas12a amplification (**b**). Conditions varied in this study to improve conventional NALFA for detection of the reporters in CRISPR/Cas12a (**c**).

In recent years, however, there has been increasing interest in nucleic acid-based POCT to overcome the inherent sensitivity limitations of immunoassays. This shift has been largely driven by advances in isothermal amplification techniques, which allow efficient amplification of DNA or RNA within 15–60 min under constant temperature conditions [10]. Methods such as recombinase polymerase amplification (RPA) and loop-mediated isothermal amplification (LAMP) offer clear advantages over conventional PCR, eliminating the need for thermocyclers and strictly controlled laboratory environments [11]. Due to their reduced equipment requirements and shorter analysis time, isothermal amplification methods are much more compatible with POCT diagnostics. A particularly promising format is the nucleic acid lateral flow assay (NALFA), which enables visual detection of the amplified products generated during isothermal amplification [12]. These systems operate relatively simply: low-molecular-weight tags (such as biotin, fluorescein, or digoxigenin) or specific single-stranded DNA fragments are introduced into the amplification mixture for subsequent recognition on the test strip. The NALFA strip contains receptors such as streptavidin, anti-tag antibodies, or complementary oligonucleotides that rapidly capture the labeled amplicons, producing visible signals in the corresponding binding zones (Figure 1a).

Beyond isothermal amplification, CRISPR (clustered regularly interspaced short palindromic repeats)/Cas12a (CRISPR-associated endonuclease Cas12a)-based detection systems have emerged as a powerful platform for nucleic acid recognition [13]. These assays exploit the exceptional specificity of the Cas12a–guide RNA (gRNA) complex, in which the gRNA contains a 20–25 nt sequence complementary to the DNA target. Upon recognition of the target sequence, Cas12a becomes activated and acquires trans-nuclease activity, enabling it to cleave any single-stranded DNA in the reaction mixture. To visualize this process, single-stranded DNA reporters containing two tags (fluorescent label/quencher for fluorescence detection, biotin/fluorescein for detection with test strips) are typically employed. In current practice, conventional lateral flow tests (LFTs) for detecting reporters in CRISPR/Cas12a reactions are constructed in the DETECTR (DNA endonuclease targeted CRISPR trans reporter) format [14,15]. Structurally, this configuration replicates the conventional NALFA design used for detecting isothermal amplification products, consisting of gold nanoparticle (GNP)–anti-fluorescein conjugates, streptavidin immobilized in the first binding zone, and protein A in the second zone (see Figure 1b). However, unlike in isothermal amplification assays, where NALFA directly captures and detects the amplified product, in CRISPR/Cas-based assays, NALFA is designed to capture uncleaved reporters in the first zone, thereby blocking the passage of the GNP conjugate to the second zone. In a positive reaction, fewer uncleaved reporters remain, reducing this blockage and allowing the conjugate to reach the second zone, which is then visualized as a positive result. From a mechanistic standpoint, this configuration poses inherent analytical challenges. The appearance of a positive signal does not require the actual product of the CRISPR/Cas12a reaction—the cleaved reporter—but rather depends on subtle kinetic competition between interacting components. Consequently, nonspecific migration of the GNP conjugate can lead to false-positive results, especially under conditions of suboptimal component balance or limited interaction time.

Although this potential limitation of CRISPR/Cas12a-LFT is rarely discussed in the literature, likely due to the limited presentation or underreporting of negative experimental outcomes, several studies attempted to modify the test architecture to detect a product specifically released upon reporter cleavage. These strategies include systems that register the release of human chorionic gonadotropin [16,17], immunoglobulin G (IgG) [18], or a branched PEG construct bearing multiple fluorescent tags [19]. A similar trouble exists in CRISPR/Cas13a systems, where NALFA has also been modified to detect DNA constructs generated after reporter cleavage [20]. However, these designs remain isolated examples and have not replaced the original DETECTR configuration. Given the rapid expansion of CRISPR/Cas-based diagnostics across diverse targets and application fields, it becomes essential to characterize the specific interactions and kinetic constraints that govern conventional LFT architectures. Such understanding forms the foundation for developing improved design strategies that enhance analytical reliability and sensitivity in CRISPR/Cas12a detection systems.

The objective of this study was to achieve a rational understanding of the limitations inherent in the standard NALFA–DETECTR configuration employing conventional dual-labeled reporters in CRISPR/Cas12a assays and identify design and operational parameters that maximize analytical performance. To this end, we performed a systematic experimental investigation encompassing 480 configurations of LFT systems, in which the concentrations and combinations of all major assay components—including the reporter—were varied. The key factors considered are summarized in Figure 1c. Complementary computational modeling was employed to interpret the observed trends and elucidate the kinetic constraints governing the interactions between assay components. Together, these approaches enabled the identification of mechanistic causes underlying false-positive outcomes and the derivation of optimization strategies that substantially improve the reliability and sensitivity of CRISPR/Cas12a-based LFT detection. The optimized system was validated using a CRISPR/Cas12a–LFT assay for the detection of *Erwinia amylovora*, the causative agent of fire blight—a major bacterial disease affecting rosaceous plants and a significant threat to global apple and pear production [21].

## 2. Materials and Methods

### 2.1. Chemicals and Materials

Primers, oligonucleotide reporters, and gRNA (sequences are presented in the Supplementary Materials (SM), Section S1, Table S1) were custom-synthesized by Lumiprobe (Moscow, Russia). Mouse anti-fluorescein antibodies (antiFAM) were obtained from Bialexa (Moscow, Russia), and streptavidin and protein A from Imtek (Moscow, Russia). EnGene LbCas12a, NEBuffer 2.1r, and Q5 polymerase were purchased from New England Biolabs (Ipswich, MA, USA). Taq polymerase, polymerase buffer, dNTPs, DNA markers for electrophoresis, and the Cleanup Standard DNA Extraction Kit were obtained from Evrogen (Moscow, Russia). The M-Sorb-Vet-DNA Extraction Kit was purchased from Syntol (Moscow, Russia). The BioMaster LAMP SYBR Kit for LAMP with SYBR Green I detection was obtained from Biolabmix (Novosibirsk, Russia). Amicon Ultra 3K filters were obtained from Merck Millipore (Burlington, MA, USA) and agarose from neoFroxx (Einhausen, Germany). Hydrogen tetrachloroaurate (III), bovine serum albumin (BSA), sodium azide, glycerol, and ethidium bromide were purchased from Sigma-Aldrich (St. Louis, MO, USA). Lateral flow membranes were sourced from Sartorius Stedim Biotech (Göttingen, Germany; nitrocellulose membrane CN-95) and Advanced Microdevices (Ambala Cantt, India; conjugate pad PT-R5, sample pad GFB-R4, absorbent pad AP045, and plastic backing LP25). Analytical-grade reagents were used for buffer preparation. Inactivated *E. amylovora* cells (CFBP 1430 strain) were provided by the All-Russian Plant Quarantine Center (Bykovo, Moscow region, Russia).

### 2.2. Synthesis of Gold Nanoparticles (GNPs)

Gold nanoparticles (GNPs) of different sizes were synthesized according to the procedure described in [22], using 1 mL of 1% HAuCl_4_ with a final reaction volume of 100 mL. The amount of 1% sodium citrate used as the reducing agent was varied: 3 mL (GNP-1) or 4.2 mL (GNP-2). Sodium citrate was added under vigorous stirring to the boiling solution of HAuCl_4_. The GNP solution was boiled for an additional 30 min, cooled to room temperature, and stored at 4 °C.

### 2.3. Synthesis of GNP–Antibody Conjugates

Both GNPs were conjugated with antiFAM antibodies via physical adsorption. The pH of GNP solutions was adjusted to 9.0 using 0.1 M K_2_CO_3_. Then, 10 µg (for GNP-1) or 12 µg (for GNP-2) of antiFAM antibodies were added per 1 mL of GNP solution. The mixture was incubated for 1 h at room temperature with continuous stirring. BSA was then added to a final concentration of 0.25%. The mixture was centrifuged at 15,000× *g* (GNP-1) or 20,000× *g* (GNP-2) for 30 min at 4 °C. The resulting GNP conjugates were resuspended in conjugate buffer (0.1 M Tris-HCl, pH 8.6, containing 1% BSA, 1% trehalose, 0.1% polyvinylpyrrolidone 25, 0.1% Tween-20, and 0.1% sodium azide).

### 2.4. Characterization of GNPs and GNP–Antibody Conjugates

GNP samples were dropped onto a 300-mesh grid (Pelco International, Fresno, CA, USA) coated with a polyvinyl formal support film. Images were acquired using a CX-100 transmission electron microscope (Jeol, Tokyo, Japan) at 80 kV. Particle size distributions were analyzed using Image Tool v. 3.0 software (University of Texas Health Science Center, San Antonio, TX, USA).

Absorption spectra were recorded using a Libra S80 spectrophotometer (Biochrom, Cambridge, UK). Hydrodynamic diameters were measured using a Zetasizer Nano (Malvern Panalytical, Malvern, UK) at 25 °C and a scattering angle of 173°.

### 2.5. Determination of Active Binding Sites for antiFAM–GNP Conjugates

The number of active antibody binding sites on the conjugates was determined via fluorescein quenching of the reporter FAM-dT10-Bio, as described by Sotnikov et al. [23] with modifications. Briefly, FAM-dT10-Bio reporter solutions (4–350 nM) were prepared in PBS containing 0.05% Triton X-100 (PBST). Mixtures of the same concentrations of reporter with GNP conjugates (A_520_ = 1 in the reaction mixture) were also prepared. Control samples contained antiFAM antibodies (10 µg/mL) instead of conjugates. The mixtures were incubated for 5, 10, 30, and 60 min, after which fluorescence intensity (λ_ex_ = 485 nm, λ_em_ = 518 nm) was recorded using a Zenyth 3100 multimode plate reader (Anthos Labtec Instruments, Salzburg, Austria), and the signal was quantified in fluorescence relative units (FRUs). Bound reporter concentration was calculated according to the formula provided in SM Section S2.

### 2.6. Preparation of Lateral Flow Test Strips for Reporter Detection

Binding reagents were dispensed onto CN-95 nitrocellulose membranes at 0.15 µL/mm in 50 mM phosphate buffer (pH 7.4) with 100 mM NaCl using an IsoFlow multichannel dispenser (Imagene Technology, Lebanon, NH, USA). Streptavidin was applied to the control zone at concentrations of 0.3–4.0 mg/mL, while protein A was applied to the test zone at 0.5 mg/mL. GNP conjugates adjusted to an optical density of A_520_ = 3.0 were applied to PT-R5 fiberglass membranes at 2 µL/mm (6 µL per strip), 1.3 µL/mm (4 µL), 1 µL/mm (3 µL), or 0.7 µL/mm (2 µL). Membranes were dried for 2 h at 37 °C and assembled on a plastic backing in two configurations:

(1) A standard composite including nitrocellulose, conjugate pad, sample pad (GFB-R4), and absorbent pad (AP045) as described in [24],

(2) A simplified composite containing only nitrocellulose and absorbent pad, designed for assays involving pre-incubation of the sample with the conjugate [25].

The composites were cut into 3 mm-wide strips using an Index Cutter-1 guillotine (A-Point Technologies, Ontario, CA, USA), sealed with desiccant, and stored at room temperature.

### 2.7. Detection of Reporters Using Lateral Flow Test Strips of Different Compositions

For strips containing a conjugate pad, test samples (100 µL PBST containing 10–500 nM reporter) were incubated with the strips for 10 min. The strips were then removed, and results were visually inspected and recorded. Digital quantification was performed by scanning the strips (Canon 9000F Mark II, Canon, Tokyo, Japan) and analyzing color band intensity using TotalLab TL120 v. 2009 software (Nonlinear Dynamics, Newcastle upon Tyne, UK). A densitometric profile was constructed for each test strip. The signal of each peak (color intensity) was calculated as peak volume per unit area and expressed in relative units (RUs). The visual limit of detection (LOD) was determined as the minimal target concentration at which the color intensity in the test zone was visible to the naked eye that corresponded to 1 RU for the used procedures of image processing according to Safenkova et al. [19]. The digital LOD was calculated using the 3σ method based on the signal for negative sample.

For strips without a conjugate pad, the sample was pre-incubated with the antiFAM–GNP conjugate for 5 min at room temperature, then the mixture was applied to the strip for 10 min. After incubation, strips were rinsed in PBST for 10 min, dried, and analyzed as described above.

### 2.8. Numerical Modeling of Interaction Kinetics in Conventional NALFA for CRISPR/Cas12a

The studied interactions in the designed LFA system were described using mathematical modeling with the COPASI 4.45 software (Build 298). Several assumptions and simplifications were adopted, consistent with previously developed LFA kinetic models [26,27]. A specific feature of our assay design is that reagents first interact in the control zone and then in the test zone, unlike conventional LFA schemes. Therefore, the binding processes in the control zone determine the signal level in the test zone. A detailed description of the model and assumptions is provided in the SM (Section S3).

### 2.9. Preparation of dsDNA Target for CRISPR/Cas12a

Total DNA was extracted from inactivated *E. amylovora* cells using the M-Sorb-Vet-DNA kit (Syntol, Moscow, Russia) according to the manufacturer’s instructions. DNA concentration was determined spectrophotometrically using a NanoDrop ND-2000 (Thermo Fisher Scientific, Waltham, MA, USA).

PCR amplification of the *hisZ* gene fragment of *E. amylovora* (sequence is presented in Figure S1, Section S1, SM) was performed according to [28] using 500 nM of each primer, 200 µM dNTP, Q5 polymerase, and 20 ng of total DNA template. The reaction (38 cycles) was carried out in a BioRad T100 thermal cycler (Hercules, CA, USA) under the following conditions: 95 °C for 30 s (denaturation), 55 °C for 30 s (annealing), and 72 °C for 60 s (extension). Amplicons were purified by electrophoresis in 2% agarose gel (20 mM Tris–acetate buffer, 0.2 mM EDTA, pH 8.3) followed by extraction using a DNA gel recovery kit (Evrogen). dsDNA concentration was measured in triplicate using NanoDrop ND-2000.

### 2.10. LAMP Amplification Prior to CRISPR/Cas12a Reaction

LAMP amplification was performed using the BioMaster LAMP SYBR Kit (Biolabmix, Novosibirsk, Russia) with SYBR Green I detection according to the manufacturer’s instructions. The reaction mixture (25 µL) contained 0.75 U Bst DNA polymerase, 50 mM Tris-HCl (pH 8.9), 10 mM KCl, 1 mM dNTP, 6 mM MgCl_2_, SYBR Green I, and 0.25% Tween 20. Two primer sets were used (see Table S1, SI). The primer set proposed by Bühlmann et al. [29], included six primers: hisZ-F3, hisZ-B3, hisZ-FIP, hisZ-BIP, hisZ-LF, and hisZ-LB. The reaction used 2 µL of 367 bp DNA amplicon as a template. LAMP reactions were incubated at 65 °C for 30–40 min. Real-time fluorescence monitoring was performed on a LightCycler 96 system (Roche, Basel, Switzerland) with excitation at 497 nm and emission at 520 nm, recorded every minute.

### 2.11. Detection of dsDNA Target with CRISPR/Cas12a

The CRISPR/Cas12a reaction was carried out using gRNA specific to the *hisZ* gene [28] (sequences in Table S1, SI). The reaction mixture was prepared by combining 60 nM gRNA and 67 nM Cas12a (NEB, Ipswich, MA, USA) in 10 mM Tris-HCl (pH 7.9) containing 50 mM NaCl, 10 mM MgCl_2_, and 100 µg/mL BSA (NEBuffer r2.1). The mixture was incubated for 10 min at 25 °C. Then, 2 µL of DNA sample (PCR amplicon or LAMP product) and the reporter were added to a total volume of 30 µL.

For fluorescence detection, ROX-dT10-BHQ2 reporter was added to a final concentration of 500 nM. For LFT detection, FAM-dT10-Bio, FAM-dT50-Bio, 3FAM-dT10-Bio, and 3FAM-dT50-Bio reporters (Table S1, SI) were used at varying concentrations.

Fluorescence assays were performed in a LightCycler 96 (Roche, Mannheim, Germany) at 37 °C with ROX fluorescence measured (λ_ex_ = 578 nm, λ_em_ = 604 nm) every minute for 30–40 min. The values that exceeded the mean value for three standard deviations for a negative sample by three standard deviations were considered to be the detection limits.

For LFT detection, the reaction mixture was incubated for 30 min at 37 °C using a TDB-120 Dry Block Thermostat (Biosan, Riga, Latvia), then mixed with an equal volume of 2× PBST containing the antiFAM–GNP conjugate (optimized according to Section 2.7). The LFT-CRISPR strip was immersed into the mixture, and the results were visually evaluated after 10 min. Digital analysis was performed as described in Section 2.7.

## 3. Results and Discussion

### 3.1. Design of Strategies to Improve of Conventional CRISPR/Cas12a–LFT System

The conventional reporter employed in CRISPR/Cas12a systems coupled with LFT detection is a single-stranded DNA oligonucleotide, typically 8 nucleotides or longer [30], carrying a biotin tag at one end and a fluorescein (FAM) tag at the other, as originally described in the DETECTR scheme [14]. In this configuration, streptavidin immobilized in the control zone binds to the biotin tag, while anti-FAM antibodies conjugated to gold nanoparticles (GNPs) interact with the fluorescein tag (see Figure 1b).

According to the DETECTR principle, in a negative reaction (no reporter cleavage), the antiFAM–GNP conjugate should be completely retained in the control zone via the formation of streptavidin–reporter–antiFAM–GNP complexes, thereby preventing the conjugate from migrating to the test zone where it could bind to protein A. To achieve this, the reporter concentration should be sufficiently high to ensure full binding of the conjugate, while the streptavidin concentration should exceed that of the reporter to efficiently capture all complexes.

However, this balance becomes critical in a positive reaction (reporter cleavage). An excessive concentration of reporter may reduce assay sensitivity, since the fraction of cleaved reporter may be too small to allow for effective migration of the conjugate to the test zone. Conversely, lowering the reporter concentration—while beneficial for signal release—can diminish the efficiency of Cas12a trans-cleavage due to substrate limitation. Thus, the optimal performance of the CRISPR/Cas12a–LFT system depends on the fine-tuning of reagent ratios and binding kinetics.

To systematically identify these dependencies, we designed a set of optimization strategies involving (Figure 1c):

(i) variation of the conjugate concentration,

(ii) adjustment of streptavidin density in the control zone,

(iii) tuning of reporter concentration, and

(iv) control of the interaction time between the conjugate and the reporter.

The number of conjugate particles—or more precisely, the number of active FAM-binding sites—can be modulated either by changing the concentration of the conjugate or by varying the carrier capacity of anti-FAM antibodies, i.e., the size of the gold nanoparticles. Both strategies were experimentally investigated to assess their effects on the sensitivity and signal intensity of the LFT response.

### 3.2. Preparation and Characterization of antiFAM–GNP Conjugates for LFT Detection of Reporters in CRISPR/Cas12a

Two GNP preparations differing in their average diameters were synthesized: GNP-1 with a diameter of 21.6 ± 3.5 nm (GNP_21_) and GNP-2 with a diameter of 16.0 ± 2.1 nm (GNP_16_). TEM images of both nanoparticle preparations and histograms based on over 100 measured particles are presented in Figure S2, Section S4, SM. Spectrophotometric analysis (absorption maximum at 520 nm for both preparations) and DLS measurements (hydrodynamic diameters of 32.1 nm for GNP_21_ and 23.5 nm for GNP_16_) confirmed the TEM data and the homogeneity of the obtained preparations (SM, Section S4).

The conjugates with antiFAM synthesized using both GNP preparations exhibited an increase in hydrodynamic diameter by 56.6 nm for GNP_21_ and 42.5 nm for GNP_16_, indicating efficient antibody immobilization on the nanoparticle surface (Figure S3, Section S4, SM). Spectrophotometric characterization of the conjugates also revealed a red shift in the absorption maximum to 530 nm for both samples (Figure S4, Section S3, SM).

**Figure 2:**
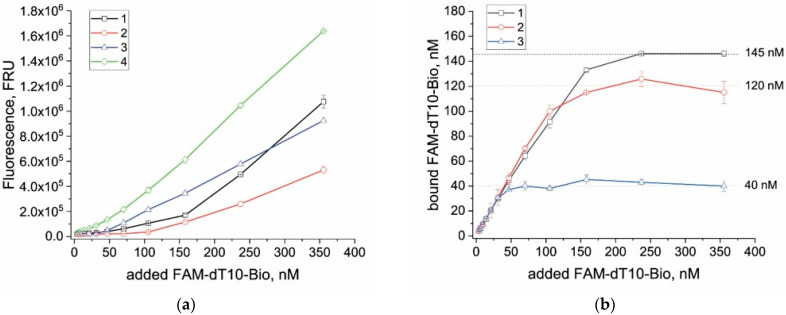
Characterization of FAM-dT10-Bio reporter binding to antiFAM sites after 10 min incubation. (**a**) Fluorescence responses for various reporter concentrations. (**b**) Dependence of reporter bound to antiFAM sites on the concentration of added reporter. Numbers indicate: addition of antibodies (10 μg/mL in the reaction mixture) (1), addition of antiFAM-GNP_16_ conjugate (A_520_ = 1 in the reaction mixture) (2), addition of antiFAM-GNP_21_ conjugate (A_520_ = 1 in the reaction mixture) (3), reporter FAM-dT10-Bio without antiFAM sites (4). The dotted lines indicate the number of active binding sites in the conjugate.

Functional characterization of the conjugates was carried out by evaluating their binding to the conventional reporter FAM-dT10-Bio using a direct fluorescence quenching approach. When antibodies bind to the fluorescent label, fluorescence quenching occurs—a phenomenon described previously in several studies [23,31]. In this work, this single-step method was purposely chosen as it allows for direct signal registration without additional separation of bound and unbound FAM-labeled reporters and provides higher accuracy and reproducibility of measurements. Similar quenching was also observed for FAM incorporated into the oligonucleotide, as confirmed by the fluorescence dependence shown in Figure 2a. At low reporter concentrations, when the molar ratio of antibody to reporter reached saturation, nearly complete fluorescence quenching was observed, indicating complete binding of fluorescein.

The same experiment was performed for both conjugates to assess reporter binding. The initial optical density (A_520_ = 1) was identical for both samples, corresponding to different nanoparticle counts in solution (calculated values are given in Table S2, Section S4, SM). The obtained results demonstrated a marked difference between the two conjugates in the amount of quenched reporter: the GNP_16_ conjugate possessed a higher number of active binding sites, corresponding to 120 nM (Figure 2b), while the GNP_21_ conjugate exhibited approximately threefold fewer binding sites—40 nM (Figure 2b). These differences are primarily attributed to the higher number of smaller particles, and consequently, a greater total surface area available for antibody immobilization (see Table S2, Section S4, SM).

Comparison of fluorescence curves obtained for the reporter with either free antibodies or GNP conjugates after 5, 10, 30, and 60 min of incubation revealed no significant differences in fluorescence quenching (Section S5, SM). Therefore, the equilibrium of the FAM–antiFAM binding interaction is reached within the first 5 min of the reaction.

### 3.3. Characterization of Reporter/LFT Systems with Different Compositions in the Absence of Cleaved Reporters (Negative Experiment)

We prepared several series of test strips differing in two parameters: the concentration of streptavidin immobilized in the test zone (ranging from 0.3 to 4 mg/mL) and the concentration of the conjugate (A_520_ = 2, 3, 4, or 6) localized on the membrane or added in an equivalent amount directly to the solution. In some cases, both conjugates were used simultaneously. In total, 60 different LFT variants were obtained, each tested with eight reporter concentrations: 500, 400, 250, 125, 62, 31, 15, and 10 nM, resulting in the evaluation of 480 LFT–reporter combinations.

The primary goal was to find the conditions under which no false-positive results occur when only the intact (uncleaved) reporter is present in solution. As noted in the Introduction, for the DETECTR-type assay, the main risk is the appearance of false-positive results because coloration of the second test zone may occur solely due to the conjugate reaching it, even in the absence of the cleaved reporter (Figure 1b). Therefore, the absence of coloration in the second test zone is a prerequisite for further testing of positive samples containing the cleaved reporter.

**Figure 3:**
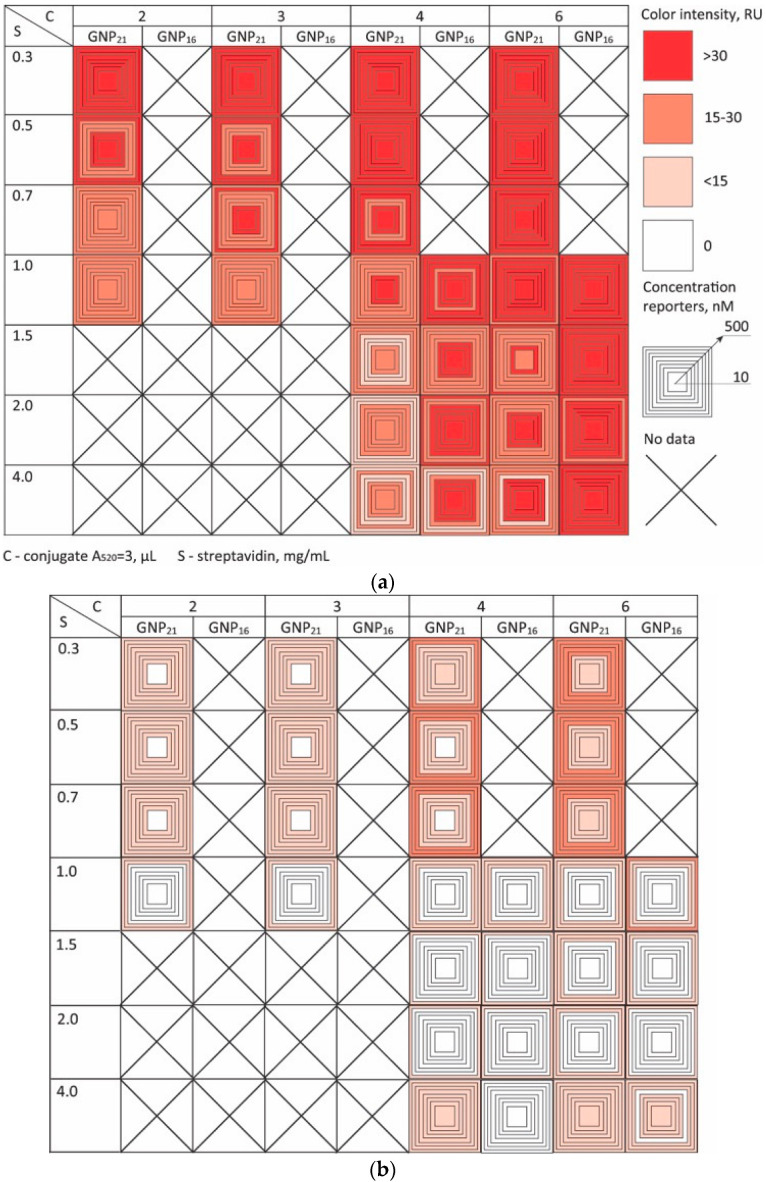

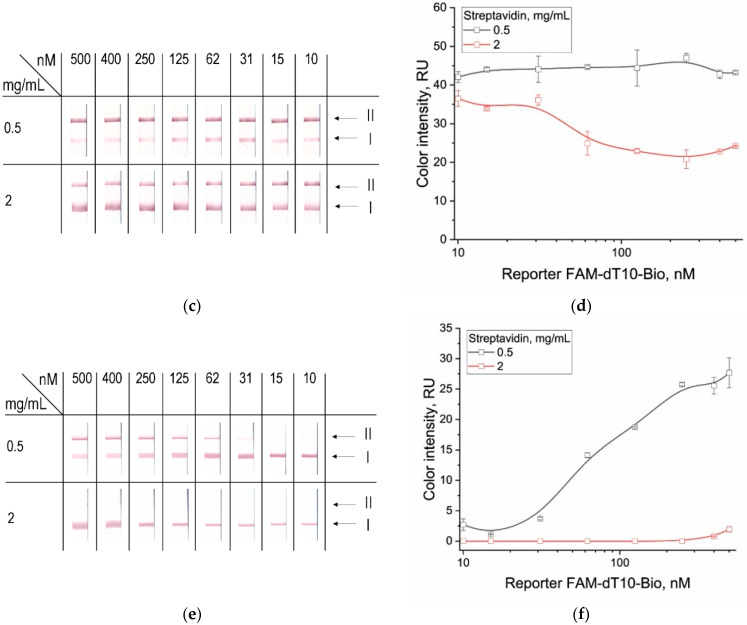
Evaluation of reporter/LFT systems of different compositions in the absence of cleaved reporters (negative experiment). (**a**) Results presented as a color gradient map, where each cell corresponds to a specific concentration of streptavidin (0.3–4 mg/mL), concentration of antiFAM-GNP conjugate (A_520_ = 3.0, 2–6 μL), conjugate type (GNP_21_ or GNP_16_) for conjugate immobilized on the membrane within the test strip. Contours within each cell indicate reporter concentration, with the direction from outer to inner contour representing sequential decrease: 500, 400, 250, 125, 62, 31, 15, 10 nM. The absence of color indicates no false-positive events, whereas shading from light pink to intense red corresponds to increasing false-positive signal. (**b**) Results presented as a color gradient map for conjugate added directly to the reporter solution and incubated for 5 min. (**c**) Scans of LFT strips at different streptavidin and reporter concentrations for antiFAM-GNP_21_ conjugate (A_520_ = 3.0, 6 μL) immobilized on the membrane. I indicates the first (control) zone, II indicates the second (test) zone. (**d**) Dependence of color intensities in the second (test) zone on reporter and streptavidin concentrations for antiFAM-GNP_21_ (A_520_ = 3.0, 6 μL) immobilized on the membrane. (**e**) Scans of LFT strips at different streptavidin and reporter concentrations for antiFAM-GNP_21_ (A_520_ = 3.0, 6 μL) added directly to the reporter solution, 5 min pre-incubation. (**f**) Dependence of color intensities in the second (test) zone on reporter and streptavidin concentrations for antiFAM-GNP_21_ (A_520_ = 3.0, 6 μL) added directly to the reporter solution, 5 min pre-incubation.

All results for 480 combinations are summarized in a gradient color map shown in Figure 3a,b, where each cell corresponds to a particular combination of streptavidin concentration, conjugate concentration, conjugate type (GNP_21_ or GNP_16_), and conjugate application method (either pre-deposited on the membrane as part of the test strip—Figure 3a or added directly to the reporter-containing solution and incubated for 5 min—Figure 3b). Each contour within a cell corresponds to a tested reporter concentration, with the direction from outer to inner contour representing decreasing reporter concentrations (from 500 to 10 nM). Thus, each contour represents unique experimental parameters for five parameters: streptavidin concentration, conjugate concentration, conjugate type (GNP_21_ or GNP_16_), conjugate application method, and reporter concentration. The absence of coloration within a contour indicates the absence of signal in the second test zone—that is, a correct negative result. Conversely, coloration of a contour indicates the presence of a false-positive signal in the second test zone.

A color scale is provided in Figure 3a,b to indicate the intensity of coloration: weak (0–15 a.u.), moderate (15–30 a.u.), and strong (>30 a.u.). Notably, all experiments using membrane-immobilized conjugates resulted in false-positive signals (Figure 3a). Typical examples of the corresponding test strips and test zone signals are shown in Figure 3c,d, while all additional test strip scans and corresponding signal values are provided in Section S6, SM.

In contrast, experiments where the conjugate was preincubated with the reporter for 5 min provided distinctly different results (Figure 3b). As the streptavidin concentration increased and the reporter concentration decreased, the coloration of the test zone disappeared (examples of test strips and corresponding signals are shown in Figure 3e,f). False-positive results for preincubated conjugates were observed only at high reporter concentrations, with lower streptavidin concentrations associated with a higher frequency of false-positive outcomes (all relevant data are presented in Section S7, SM).

It is worth noting that no significant differences were observed between conjugates containing gold nanoparticles of different sizes in negative tests, indicating that particle size did not affect nonspecific binding or background signal formation. Based on these results, several optimized test configurations were selected for further evaluation with positive samples. These configurations employed streptavidin at 2 mg/mL and the conjugate solution at a concentration corresponding to A_520_ = 3.0 (6 µL per test). All selected systems demonstrated a complete absence of false-positive signals.

### 3.4. Evaluation of Interaction Processes in the LFT by Numerical Modeling

Before proceeding to the testing of positive samples, we sought to interpret the observed false-positive results. To this end, mathematical modeling of the affinity complex formation processes was performed to assess the influence of various factors on the analytical outcome. A numerical model of the analytical system was developed using the COPASI v. 4.45 software environment (see detailed description in Section S3, SM).

The studied LFT scheme involves sequential interactions—first in the control zone, followed by the analytical (test) zone. In the case of a negative result, all antiFAM–GNP conjugates should bind in the control zone, whereas in a positive test, a portion of the conjugate passes through to the test zone and becomes captured there. Therefore, in this assay format, the control zone affects the signal in the test zone. In the absence of analyte, the control zone should capture the maximum possible amount of conjugate to prevent background coloration in the test zone. To simulate the appropriate conditions for interactions, we used the main experimental parameters: streptavidin concentration equal to 1 mg/mL, conjugate concentration equal to the antiFAM sites for the 6 μL of antiFAM-GNP_16_ conjugate, reporter concentration in the range from 0.1 to 500 nM, where 0.1–1 nM can be considered as the concentration of uncleaved reporter in a positive reaction.

**Figure 4:**
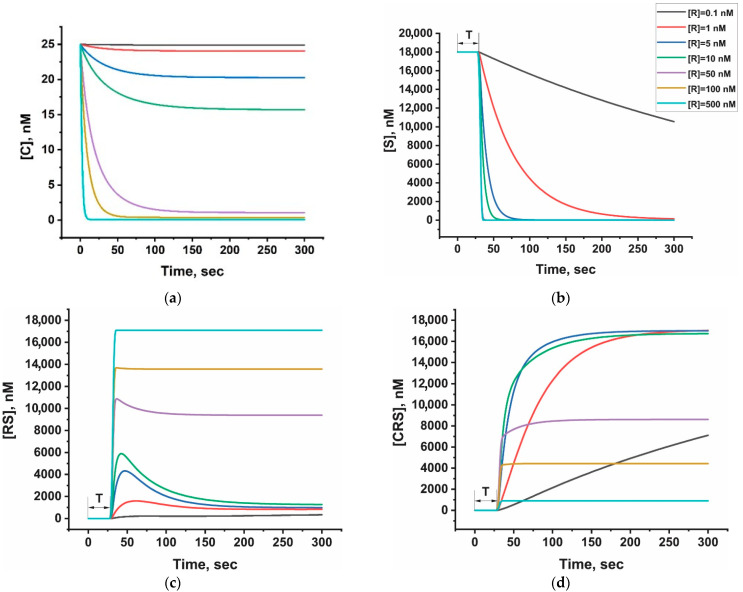
Modeled kinetic curves of concentration changes of main components in the reaction mixture (COPASI) at different reporter concentrations ([R]). Initial concentrations: [C]_0_ = 2.5 × 10^−8^ M (active conjugate site concentration determined from experimental data, see Section 3.1) and [S]_0_ = 1.8 × 10^−5^ M (streptavidin concentration). (**a**) AntiFAM-GNP conjugate ([C]). (**b**) Immobilized streptavidin ([S]). (**c**) Reporter-streptavidin complex ([RS]). (**d**) Colored complex in the control zone ([CRS]). T denotes the time required for the liquid front to reach the control zone.

Figure 4 presents theoretical kinetic curves describing the changes in reagent concentrations from the start of the interaction between the nanoparticle conjugate (C) and the reporter (R). As component R is added to the sample, the starting point of the analysis corresponds to the contact between R and C. Here, T denotes the time required for the liquid front to reach the control zone which, according to experimental data, corresponds to approximately 30 s. Consequently, reactions in the control zone begin only after this interval.

As shown in Figure 4a, at high reporter concentrations (R), the available antiFAM binding sites are rapidly saturated. Despite the substantially higher concentration of streptavidin (S), its consumption occurs even more rapidly (Figure 4b) because streptavidin is localized only within the control zone, while R is dissolved throughout the sample volume and continuously supplied to the reaction area with the fluid flow. Moreover, the biotin–streptavidin interaction is characterized by very high affinity. As a result, C is quickly converted to CR, and S to RS (reporter–streptavidin complex), both of which can no longer interact with each other. Consequently, CR freely passes through the control zone without forming the final complex CRS (conjugate–reporter–streptavidin) (Figure 4d). This CR fraction subsequently binds in the analytical zone, leading to a false-positive result (see Figure 3a, cells corresponding to high reporter concentrations).

Figure 4d also demonstrates that optimal results are achieved when the reporter concentration [R]_0_ (5, 10 nM) is lower than the conjugate concentration [C]_0_ (25 nM). However, if [R]_0_ is lower by more than an order of magnitude, the rate of formation of the colored CRS complex is significantly reduced, while the [C]_0_ concentration remains consistently high. This creates the conditions for C to overshoot the control zone and stain the test zone in the absence of analyte.

The model also suggests that false-positive signals can be prevented either by using significantly higher streptavidin concentrations—although this is limited by the capacity of the membrane (at streptavidin concentrations of 4 mg/mL, binding efficiency decreases noticeably; see Figure 3a,b, Figures S12 and S27 SM)—or by reducing the reporter concentration [R]_0_. However, if [R]_0_ is much lower than [C]_0_, substantial concentrations of free antiFAM sites in the conjugate [C] will remain in the reaction medium (Figure 4a). These sites can participate in the formation of colored complexes upon interaction with RS, but this requires sufficient accumulation of RS in the control zone.

As shown in Figure 4c, the concentration of RS exhibits a transient maximum, which is more pronounced at lower [R]_0_ values: RS initially accumulates and is then depleted through the reaction RS + C → CRS, producing a transient peak. At higher initial reporter concentrations (R_0_), free C becomes limited due to the reaction C + R → CR, which suppresses the overshoot. Therefore, the kinetics of complex formation should also be considered, as free conjugate may pass through the control zone before sufficient complexation occurs, again leading to false-positive results. This effect is consistent with experimental observations (Figure 3a), where false-positive results were obtained for systems using membrane-localized conjugates—C did not have sufficient time to react with either R or RS. Preincubation of the conjugate with the reporter for 5 min effectively ensures CR complex formation. Alternatively, this issue can also be mitigated by using membranes with smaller pore sizes, which slow down sample flow and enhance reaction efficiency. The identified trends provide a mechanistic basis for interpreting the experimental results obtained for both negative and positive reactions. However, the model is intended primarily to support and rationalize the proposed LFT configuration rather than to serve as a tool for precise quantitative prediction.

### 3.5. Detection of dsDNA Targets in the CRISPR/Cas12a System with LFT Reporter Readout

The selected LFT configurations (streptavidin, 2 mg/mL; conjugate in solution, A_520_ = 3, 6 μL) were evaluated using positive CRISPR/Cas12a reactions. As a target, we selected the *hisZ* gene of *Erwinia amylovora*, a species-specific target frequently used in PCR- and isothermal amplification-based diagnostic assays [29]. A purified PCR product corresponding to a 367 bp dsDNA fragment (electrophoretic analysis shown in Section S8, SM) was used as the dsDNA target recognized by the designed gRNA, which activates the trans-nuclease activity of Cas12a.

**Figure 5:**
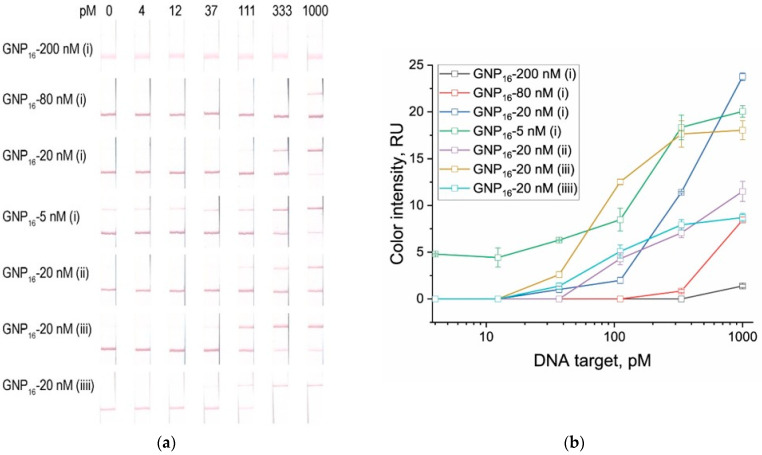

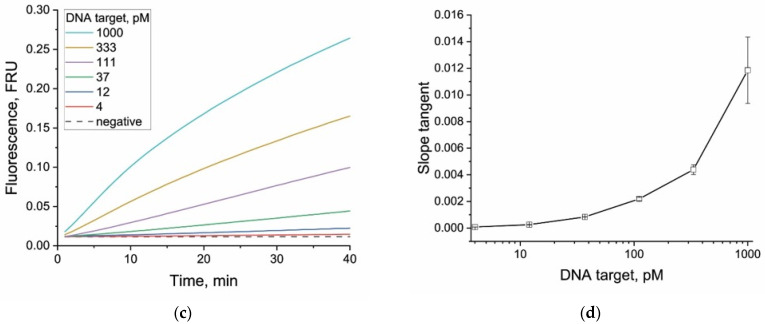
Detection of dsDNA-target (367 bp fragment corresponding to *hisZ* gene of *E. amylovora*) at various concentrations (4, 12, 37, 111, 333, 1000 pM) in CRISPR/Cas12a reactions. (**a**) Test strips (streptavidin 2 mg/mL, antiFAM-GNP_16_ conjugate in solution A_520_ = 3.0, 6 μL) after CRISPR/Cas12a assay with different reporters (i/ii/iii/iv): FAM-dT10-Bio (5, 20, 80, 200 nM)/FAM-dT50-Bio (20 nM)/3FAM-dT50-Bio (20 nM)/3FAM- dT10-Bio (20 nM). (**b**) Concentration dependences of color intensities (test zone LFT) after CRISPR/Cas12a assay. (**c**) Fluorescence curves for ROX-dT10-BHQ2 reporter in CRISPR/Cas12a assay. (**d**) Concentration dependence of fluorescence intensity of cleaved ROX-dT15-BHQ2 at 40 min.

The conventional reporter FAM-dT10-Bio at concentrations ranging from 15 to 600 nM (corresponding to 5–200 nM in the LFT sample after dilution) was tested in CRISPR/Cas12a reactions containing various dsDNA target concentrations (1000–4 pM). The results revealed pronounced differences in the concentration-dependent response profiles (Figure 5a,b). For the antiFAM–GNP_16_ conjugate, decreasing the reporter concentration from 200 to 20 nM improved the limit of detection (LOD) from 1000 to 70 pM. This suggests that a reporter concentration of 20 nM in the LFT provides the minimal amount required for complete conjugate capture in the first (control) zone. Even partial cleavage of the reporter at low dsDNA target concentrations is sufficient to disrupt the streptavidin–reporter complex, allowing part of the conjugate to migrate to the test zone and generate a positive signal. Further reduction of the reporter concentration to 5 nM led to a lower LOD of 30 pM but was accompanied by false-positive signals (Figure 5a,b).

A similar trend was observed for the antiFAM–GNP_21_ conjugate: lower reporter concentrations resulted in improved sensitivity (Section S9, SM). However, for the larger GNP_21_ nanoparticles, the LOD was consistently higher across all reporter concentrations. We hypothesize that larger nanoparticles, possessing more antiFAM binding sites per particle, require a greater number of cleaved reporter molecules to achieve the threshold shift necessary for conjugate migration to the test zone—corresponding to higher dsDNA target concentrations.

Comparison of the conjugates suggested that a reporter capable of blocking multiple binding sites might further reduce the detection limit. Therefore, for the antiFAM–GNP_16_ conjugate, we tested modified reporters with increased numbers of fluorescent labels on one end—3FAM-dT10-Bio and 3FAM-dT50-Bio (see Table S1, SI)—and, as a control, a single-labeled reporter with an extended oligonucleotide sequence, FAM-dT50-Bio. The lowest LOD (20 pM) and highest test-zone signal intensity were obtained for the 3FAM-dT50-Bio reporter, confirming the hypothesis that increasing the number of fluorescent moieties on a single reporter enhances detection sensitivity (Figure 5a,b).

Overall, the observed LOD differences, depending on the conjugate type and reporter concentration, exceeded 50-fold. We believe that such a difference in sensitivity between optimized and non-optimized CRISPR/Cas12a–LFT configurations is particularly important for amplification-free CRISPR/Cas12a test systems, which include a wide range of approaches and do not involve preliminary amplification of target DNA copies [32,33,34]. For comparison, CRISPR/Cas12a reactions with fluorescence readout were performed using the standard fluorophore–quencher probe ROX-dT10-BHQ2 (see Table S1, SI), corresponding to the canonical DETECTR scheme. The fluorescence-based LOD was 10 pM (Figure 5c,d), which is twofold lower than that achieved by LFT detection with 3FAM-dT50-Bio and sevenfold lower than with the conventional FAM-dT10-Bio reporter.

### 3.6. Applications of CRISPR/Cas12a–LFT Using the Conventional Reporter

The obtained results demonstrate that even after multifactor optimization, the conventional DETECTR-type system using the FAM-dT10-Bio reporter operated less efficiently than both fluorescence-based detection and the improved multi-labeled reporter (3FAM-dT50-Bio). Furthermore, despite proper optimization, the DETECTR LFT scheme remained highly sensitive to the balance between components, making it prone to false-positive results (see Section 3.3) or reduced sensitivity (see Section 3.5). In contrast, in fluorescence-based DETECTR assays, the signal arises exclusively from the cleaved reporter, eliminating these risks.

This highlights the importance of developing alternative CRISPR/Cas12a–LFT configurations in which the test strip is specifically designed to detect the product released after reporter cleavage [16,17,18,19]. Nevertheless, there are two areas where the DETECTR format can be applied with maximum benefit:

(1) membranes that provide a slow flow rate increase the interaction time of the components and thus minimizing false positives;

(2) in CRISPR/Cas12a–LFT coupled with prior isothermal amplification, the high abundance of dsDNA amplicons renders the assay less dependent on reporter concentration.

**Figure 6:**
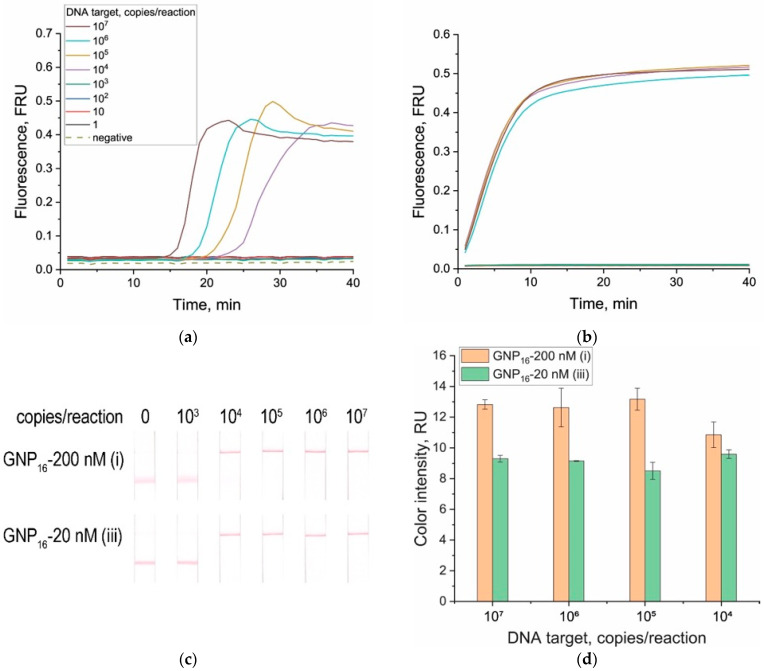
Detection of dsDNA-target (367 bp fragment corresponding to *hisZ* gene of *E. amylovora*) at different concentrations after LAMP. (**a**) Fluorescence curves for LAMP with SYBR Green I detection. (**b**) Fluorescence curves for LAMP–CRISPR/Cas12a with ROX-dT10-BHQ2 reporter. (**c**) Test strips (streptavidin 2 mg/mL, antiFAM-GNP16 conjugate in solution A_520_ = 3.0, 6 μL) after CRISPR/Cas12a assay with reporters (i/iii): FAM-dT10-Bio (200 nM)/3FAM-dT50-Bio (20 nM). (**d**) Dependence of second (test) zone color intensities on sample after CRISPR/Cas12a assay with different reporters.

The first assumption is confirmed by theoretical calculations made in Section 3.4. The second assumption was experimentally verified using a LAMP–CRISPR/Cas12a–LFT system targeting the same dsDNA sequence. The LAMP amplification alone achieved a detection limit of 10^4^ copies per reaction (Figure 6a). At high target DNA concentrations, the fluorescence curves showed an overshoot, likely caused by the formation of large concatemeric and looped DNA structures typical of LAMP. At early stages, rapid accumulation of double-stranded DNA leads to signal growth, whereas at later stages, structural rearrangements may reduce the fraction of SYBR GREEN-accessible duplex regions, resulting in a decrease toward the plateau. Subsequent CRISPR/Cas12a reactions with fluorescence detection produced strong signals for all positive samples (Figure 6b). As seen in Figure 6b, the fluorescence signals were nearly identical across all positive concentrations and substantially higher than those in Figure 5c, where no preamplification was used. This confirms that the dsDNA target concentration after LAMP greatly exceeds 1000 pM—the highest tested in Figure 5.

LAMP–CRISPR/Cas12a–LFT assays were performed for both the most effective (3FAM-dT50-Bio, 20 nM) and least effective (FAM-dT10-Bio, 200 nM) reporters. In both cases, strong positive signals were observed in the test zone (Figure 6c,d). These results confirm that when preamplification is applied, the optimization of LFT parameters and reporter composition becomes largely irrelevant due to the high dsDNA target concentration in the amplified sample. Nevertheless, even in amplified systems, a more sensitive configuration remains advantageous, especially at the very early stages of amplification when the amplicon concentration is still low, enabling rapid detection. In amplification-free CRISPR/Cas12a assays, choice of the LFT system design and reporter configuration is the key factor determining analytical sensitivity. Moreover, optimization of reporter/LFT systems without cleavage (negative experiment) is essential for both amplified and amplification-free formats for finding conditions that eliminate false-positive signals (Figure 3). Pre-incubation of the reporter with the conjugate was shown to significantly reduce the probability of false-positive signals. Importantly, the proposed workflow (LAMP–CRISPR/Cas12a–5 min incubation with the conjugate–LFT) differs from the conventional LAMP–CRISPR/Cas12a–LFT protocol only by an additional 5 min incubation step, which does not substantially increase the overall assay time. Moreover, the required additional manipulation is minimal, as the LFT buffer (already containing the conjugate) can be added directly to the reaction mixture after completion of the CRISPR/Cas12a reaction. Furthermore, implementation of a one-pot format, considered, in particular, for LAMP–CRISPR/Cas12a reactions in the review by Shi et al. [35], could further simplify handling, reduce the analysis time, and bring the system closer to a true POCT format.

## 4. Conclusions

CRISPR/Cas12a systems with LFT detection in the DETECTR format were thoroughly investigated using both experimental and computational approaches. Based on the principles of LFT detection of intact and Cas12a-cleaved reporters, we hypothesized a high probability of false-positive reactions in this system, as well as a strong dependence on the balance of interacting components. Experimental results confirmed this hypothesis by showing false-positive signals in schemes characterized by short interaction times. Numerical modeling of the interaction processes in LFT further supported this conclusion, revealing kinetic limitations at interaction times shorter than a few minutes, which significantly affect the detectable signal.

The fundamental cause of false-positive results in the studied format is that the cleaved reporter initiating the positive signal in the test zone is not an essential component for signal formation. Insufficient interaction time acts as a condition that revealed this root cause; therefore, we found that a 5 min pre-incubation of the conjugate with the reporter before LFT detection effectively compensates for these temporal limitations.

The strong dependence on the balance of interacting components was also confirmed experimentally, as the sensitivity of the system varied by more than 50-fold depending on the test system configuration. The lowest limit of detection (20 pM, *hisZ* gene of *E. amylovora*) was achieved with a configuration including streptavidin at a concentration of 2 mg/mL, an antiFAM-GNP_16_ conjugate in solution (A_520_ = 3.0, 6 μL), and a reporter containing three fluorescent labels (3FAM-dT50-Bio, 20 nM). We do not claim that the specific concentrations of the reporter and conjugate identified in this work are universally applicable to all systems. Rather, our study establishes a general optimization strategy that was validated for using bioreceptors, GNP, and membranes and can be transferred (as an approach rather than as fixed numerical values) to other test systems.

At the same time, for test systems employing prior isothermal amplification—which increases the number of dsDNA target copies—the key influencing factor is only the incubation time. Due to the high concentration of dsDNA amplicons, even less efficient configurations perform effectively. Therefore, the results obtained here are particularly important for CRISPR/Cas12a amplification-free approaches.

## Data Availability

The data presented in this study are available on request from the corresponding author.

## Supplementary Materials

The following supporting information can be downloaded at: https://www.mdpi.com/article/10.3390/bios15120812/s1, Figure S1: Sequence of *hisZ* gene (top strand in the direction from 5′ to 3′) and corresponding sequences of PCR primers (blue color), LAMP primers (black color), gRNA (red color); Figure S2: Characterization of the synthesized GNPs by TEM; Figure S3: Distribution of hydrodynamic diameters (D_H_) of GNPs and antiFAM-GNP conjugates; Figure S4: Spectra of GNPs and antiFAM-GNP conjugates (optical path length = 1 mm); Figure S5: Characterization of FAM-dT10-Bio reporter binding to antiFAM sites at different incubation time; Figure S6: Testing of reporter/LFT systems of different compositions with antiFAM-GNP_21_ conjugate immobilized on the membrane and 0.3 mg/mL streptavidin; Figure S7: Testing of reporter/LFT systems of different compositions with antiFAM-GNP_21_ conjugate immobilized on the membrane and 0.5 mg/mL streptavidin; Figure S8: Testing of reporter/LFT systems of different compositions with antiFAM-GNP_21_ conjugate immobilized on the membrane and 0.7 mg/mL streptavidin; Figure S9: Testing of reporter/LFT systems of different compositions with antiFAM-GNP_21_ conjugate immobilized on the membrane and 1.0 mg/mL streptavidin; Figure S10: Testing of reporter/LFT systems of different compositions with antiFAM-GNP_21_ conjugate immobilized on the membrane and 1.5 mg/mL streptavidin; Figure S11: Testing of reporter/LFT systems of different compositions with antiFAM-GNP_21_ conjugate immobilized on the membrane and 2.0 mg/mL streptavidin; Figure S12: Testing of reporter/LFT systems of different compositions with antiFAM-GNP_21_ conjugate immobilized on the membrane and 4.0 mg/mL streptavidin; Figure S13: Testing of reporter/LFT systems of different compositions with antiFAM-GNP_16_ conjugate immobilized on the membrane and 1.0 mg/mL streptavidin; Figure S14: Testing of reporter/LFT systems of different compositions with antiFAM-GNP_16_ conjugate immobilized on the membrane and 1.5 mg/mL streptavidin; Figure S15: Testing of reporter/LFT systems of different compositions with antiFAM-GNP_16_ conjugate immobilized on the membrane and 2.0 mg/mL streptavidin; Figure S16: Testing of reporter/LFT systems of different compositions with antiFAM-GNP_16_ conjugate immobilized on the membrane and 4.0 mg/mL streptavidin; Figure S17: Testing of reporter/LFT systems of different compositions with antiFAM-GNP_21_ conjugate added directly to the reporter solution with 5 min pre-incubation and 0.3 mg/mL streptavidin; Figure S18: Testing of reporter/LFT systems of different compositions with antiFAM-GNP_21_ conjugate added directly to the reporter solution with 5 min pre-incubation and 0.5 mg/mL streptavidin; Figure S19: Testing of reporter/LFT systems of different compositions with antiFAM-GNP_21_ conjugate added directly to the reporter solution with 5 min pre-incubation and 0.7 mg/mL streptavidin; Figure S20: Testing of reporter/LFT systems of different compositions with antiFAM-GNP_21_ conjugate added directly to the reporter solution with 5 min pre-incubation and 1.0 mg/mL streptavidin; Figure S21: Testing of reporter/LFT systems of different compositions with antiFAM-GNP_21_ conjugate added directly to the reporter solution with 5 min pre-incubation and 1.5 mg/mL streptavidin; Figure S22: Testing of reporter/LFT systems of different compositions with antiFAM-GNP_21_ conjugate added directly to the reporter solution with 5 min pre-incubation and 2.0 mg/mL streptavidin; Figure S23: Testing of reporter/LFT systems of different compositions with antiFAM-GNP_21_ conjugate added directly to the reporter solution with 5 min pre-incubation and 4.0 mg/mL streptavidin; Figure S24: Testing of reporter/LFT systems of different compositions with antiFAM-GNP_16_ conjugate added directly to the reporter solution with 5 min pre-incubation and 1.0 mg/mL streptavidin; Figure S25: Testing of reporter/LFT systems of different compositions with antiFAM-GNP_16_ conjugate added directly to the reporter solution with 5 min pre-incubation and 1.5 mg/mL streptavidin; Figure S26: Testing of reporter/LFT systems of different compositions with antiFAM-GNP_16_ conjugate added directly to the reporter solution with 5 min pre-incubation and 2.0 mg/mL streptavidin; Figure S27: Testing of reporter/LFT systems of different compositions with antiFAM-GNP_16_ conjugate immobilized on the membrane and 4.0 mg/mL streptavidin; Figure S28. Visualization of PCR products (DNA fragment of *hisZ* gene with length of 367 bp) after electrophoresis in 2% agarose gel for the *E. amylovora*; Figure S29. Detection of dsDNA-target (367 bp fragment corresponding to *hisZ* gene of *E. amylovora*) at various concentrations (4, 12, 37, 111, 333, 1000 pM) in CRISPR/Cas12a reactions; Table S1: Sequences of the primers, and reporters used in this research; Table S2: Comparative characteristics of gold nano-particles; Section S1: DNA-targets, primers, and reporters; Section S2: Determination of anti-body-bound FAM-dT10-Bio reporter; Section S3: Simulation of LFT interactions using COPASI software; Section S4: Characterization of gold nanoparticles (GNPs) and their conjugates with anti-fluorescein antibodies (antiFAM); Section S5: Characterization of FAM-dT10-Bio reporter binding to antiFAM sites at different incubation time; Section S6: Testing of reporter/LFT systems of different compositions with antiFAM-GNP conjugate immobilized on the membrane in the absence of cleaved reporters (negative experiment); Section S7: Testing of reporter/LFT systems of different compositions with antiFAM-GNP conjugate added directly to the reporter solution with 5 min pre-incubation in the absence of cleaved reporters (negative experiment); Section S8: Characterization of dsDNA target of Erwinia amylovora obtained by PCR; Section S9: Detection of dsDNA-target in CRISPR/Cas12a with LFT detection; References [36,37] are cited in the supplementary materials.
