# Supplementary material for: Advancing Lateral Flow Detection in CRISPR/Cas12a Systems Through Rational Understanding and Design Strategies of Reporter Interactions
Source: Biosensors (Basel). 2025 Dec 13;15(12):812. doi: 10.3390/bios15120812 (PMC12730283; doi:10.3390/bios15120812)
Supplement: Supplementary file 1 [file biosensors-15-00812-s001.zip › biosensors-3992330-supplementary.pdf]

Supplementary Information

Advancing Lateral Flow Detection in CRISPR/Cas12a Systems Through Rational Understanding and Design Strategies of Reporter Interactions

Irina V. Safenkova [1,*], Maria V. Kamionskaya [1], Dmitriy V. Sotnikov [1], Sergey F. Biketov [2], Anatoly V. Zherdev [1] and Boris B. Dzantiev [1]

[1] A.N. Bach Institute of Biochemistry, Research Centre of Biotechnology of the Russian Academy of Sciences, 119071 Moscow, Russia; mv.kamionskaya@fbras.ru (M.V.K.); sotnikov-d-i@mail.ru (D.V.S.); zherdev@inbi.ras.ru (A.V.Z.); dzantiev@inbi.ras.ru (B.B.D.)

[2] State Research Center for Applied Microbiology & Biotechnology, 142279 Obolensk, Russia; biketov@mail.ru

* Correspondence: safenkova@inbi.ras.ru; Tel./Fax: +7-495-954-2804

Contents

Section S1. DNA-targets, primers, and reporters 2

Section S2. Determination of antibody-bound FAM-dT10-Bio reporter 4

Section S3. Simulation of LFT interactions using COPASI software 5

Section S4. Characterization of gold nanoparticles (GNPs) and their conjugates with anti-fluorescein antibodies (antiFAM)....9

Section S5. Characterization of FAM-dT10-Bio reporter binding to antiFAM sites at different incubation time 11

Section S6. Testing of reporter/LFT systems of different compositions with antiFAM-GNP conjugate immobilized on the membrane in the absence of cleaved reporters (negative experiment)....12

Section S7. Testing of reporter/LFT systems of different compositions with antiFAM-GNP conjugate added directly to the reporter solution with 5 min pre-incubation in the absence of cleaved reporters (negative experiment)....23

Section S8. Characterization of dsDNA target of *Erwinia amylovora* obtained by PCR 34

Section S9. Detection of dsDNA-target in CRISPR/Cas12a with LFT detection....35

References 36

Section S1. DNA-targets, primers, and reporters

Table S1. Sequences of the primers, and reporters used in this research

#	Name	Sequence 5′-3′	Purpose
1	hisZ-PCR-F	TAGATAAATCAATTAAAGGCCA	Primers for PCR synthesis of dsDNA-target (fragment with 367 bp length corresponded to *hisZ* gene) of *Erwinia amylovora*, [1]
2	hisZ-PCR-R	GTCTACCTTAAATCCTTACTGC	
3	hisZ-F3	TCAAGATCGTGTGGCTATG	Primers for LAMP of *E. amylovora*, [2]
4	hisZ-B3	CTAAAAACCGGGGCAAAC	
5	hisZ-FIP	ACGATTCTACCCTTCCTGTCTACTTCTCTG GGGTTTCAGTC	
6	hisZ-BIP	ATGTCACCTGATTCTACAGCCGCAATCATTCATGGTTCTGGAC	
7	hisZ-LF	ATTAGCGGCCCGACCAA	
8	hisZ-LB	GTTAAGATGGCATGCAGA	
9	hisZ-gRNA	UAAUUUCUACUAAGUGUAGAUGGUGAUAAAACCUUUGAAUU*	gRNA recognizing dsDNA-target *hisZ* of *E. amylovora*, [1]
10	ROX-dT10-BHQ2	[ROX]TTTTTTTTTT[BHQ2]	Conventional reporter for CRISPR/Cas12a assay with fluorescence detection
11	FAM-dT10-Bio	[FAM]TTTTTTTTTT[Bio]	Conventional reporter for CRISPR/Cas12a assay with lateral flow test (LFT) detection
12	FAM-dT50-Bio	[FAM]TTTTTTTTTTTTTTTTTTTTTTTTTTTTTTTTTTTTTTTTTTTTTTTTTT[Bio]	Improved reporters for CRISPR/Cas12a assay with LFT detection
13	3FAM-dT10-Bio	([FAM][HEg])$_3$[TREBLER]TTTTTTTTTT[Bio]	
14	3FAM-dT50-Bio	([FAM][HEg])$_3$[TREBLER]TTTTTTTTTTTTTTTTTTTTTTTTTTTTTTTTTTTTTTTTTTTTTTTTTT[Bio]	

The abbreviations in the table mean [Bio] – bioitin, [FAM]—fluorescein, ROX – 5-carboxyrhodamine-X, BHQ2 – black hole quencher2, HEg – hexaethylene glycol, [TREBLER]—branch point in 5′-terminal direction due to using trebler phosphoramidite for synthesis of oligonucleotides.

* Site of recognition is denoted by the single underline

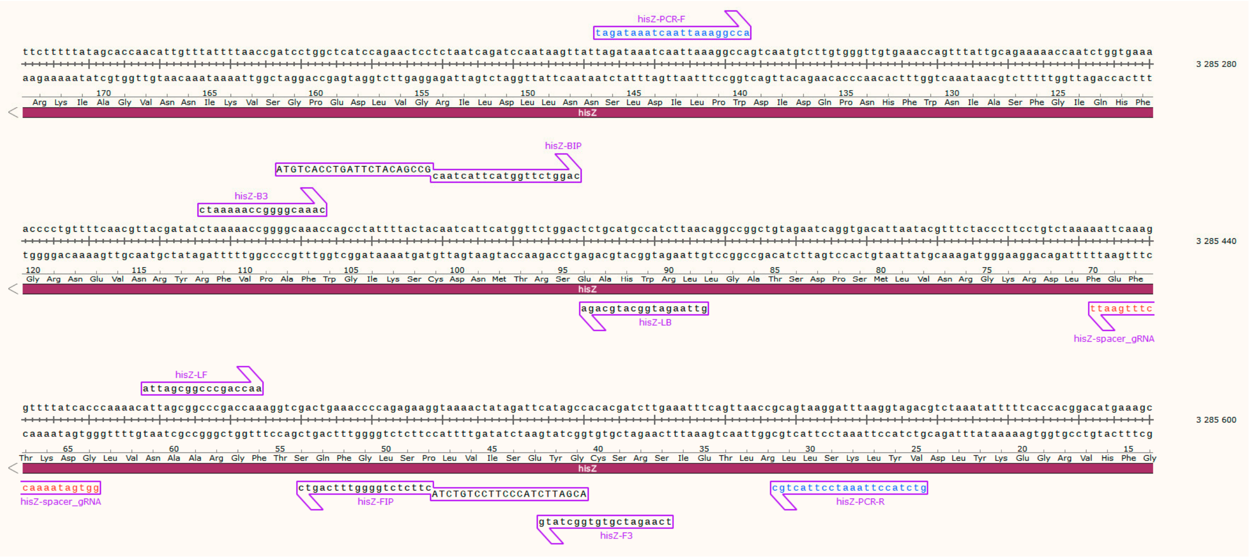

Figure S1. Sequence of *hisZ* gene (top strand in the direction from 5′ to 3′) and corresponding sequences of PCR primers (blue color), LAMP primers (black color), gRNA (red color)

Section S2. Determination of antibody-bound FAM-dT10-Bio reporter

The concentrations of antibody-bound FAM-dT10-Bio reporter (C_b) were calculated based on equation proposed by Sotnikov et al. [3]:

$F = F_1 \times C_f + 0.04 \times F_1 \cdot \times C_b$,

where F is total fluorescence minus the background;

C_f is concentrations of free FAM-dT10-Bio reporter;

F_1 is fluorescence of the unit concentration of the fluorophore that means F is equal to F_1 multiplied by its concentration (C);

coefficient 0.04 means that bound FAM-dT10-Bio is 4% of the fluorescence of free FAM-dT10-Bio in the same concentration.

Therefore,

$C_b = (F - F_1 \times C) / 0.96 \times F_1$

Section S3. Simulation of LFT interactions using COPASI software

Simplifications adopted in the model

1. The reactions are considered within two distinct compartments: the liquid sample compartment and the reaction zone compartment. Substances dissolved in the liquid sample move uniformly with the flow and are not retained by the membranes of the test strip.

2. All reagents are uniformly distributed within their respective compartments, and the reactions between them are not diffusion-limited.

3. The influence of polyvalent interactions is assumed to be negligible and is therefore not considered in the description of affinity complex formation.

4. Each affinity interaction is characterized by its averaged binding constant, which remains unchanged over time.

In a simplified representation, four affinity reactions occur sequentially in the system. Initially, the liquid sample contains reporter molecules (R) labeled with fluorescein and biotin, and antibodies against fluorescein (anti-FAM), conjugated to gold nanoparticles (C). It should be noted that the concentration [C] reflects the actual concentration of active anti-FAM sites in the conjugate and does not correspond to the total antibody concentration. This discrepancy arises because antibodies are bivalent, and, moreover, antibody immobilization on nanoparticles results in a substantial inactivation of anti-FAM sites [4].

An interaction occurs between the conjugate and the reporter according to the following scheme:

C + R ⟷ CR (reaction)

This reaction takes place in the liquid sample compartment and is initiated upon mixing of the reporter with the nanoparticle conjugate. The duration of this reaction is denoted as *t*. Subsequently, the reaction mixture migrates along the membranes of the test strip and reaches the functional zone containing immobilized streptavidin (S) after a time T. When the liquid front reaches the streptavidin zone, three additional reactions are initiated:

CR+ S ⟷ CRS (reaction 1)

R+ S ⟷ RS (reaction 2)

RS + C ⟷ CRS (reaction 3)

he species C, R, and their complex CR pass through the functional zone, while S, RS, and CRS remain confined within it, as they cannot migrate with the flow. The formation of the CRS complex leads to the accumulation of nanoparticles in the zone, which is visualized as a colored line. Therefore, the intensity of coloration is determined by the concentration of [CRS], in accordance with reactions (1) and (3). The duration of reactions (1)–(3) is *(t* – T*)*. In addition to concentrations and time parameters, the reaction rates are defined by the association (ka) and dissociation (kd) constants of each reaction.

Parameters used for numerical simulation in COPASI:

>Model

>Biochemical

>Compartments

>Reporter

Details: Simulation Type – fixed

Contained Species – C, R, CR

>Control zone

Details: Simulation Type – fixed

Contained Species – C, R, CR, S, RS, CRS

> Species

Name	Simulation Type	Initial Concentration, M	Expression (M)
R	ode	1×10^{-8}*	-(reaction).k1 × [C] × [R]+(reaction).k2 × [CR]
C	ode	2.5×10^{-8}*	-(reaction).k1 × [C] × [R]+(reaction).k2 × [CR]
CRS	ode	0	((reaction_1).k1 × [CR] × [S]-(reaction_1).k2 × [CRS]+(reaction_3).k1 × [RS] × [C]-(reaction_3).k2 × [CRS]) × Values[h]
RS	ode	0	((reaction_2).k1 × [R] × [S]-(reaction_2).k2 × [RS]-(reaction_3).k1 × [RS]*[C]+(reaction_3).k2 × [CRS]) × Values[h]
S	ode	1.8×10^{-8}*	(-(reaction_1).k1 × [CR] × [S]+(reaction_1).k2 × [CRS]-(reaction_2).k1 × [R] × [S]+(reaction_2).k2 × [RS]) × Values[h]
CR	ode		(reaction).k1 × [C] × [R]-(reaction).k2 × [CR]

*Data were determined from experimental data

>Reactions:

>Reaction

C + R = CR

Rate Law: Mass action (reversible)

>Reaction 1

S + CR = CRS

Rate Law: Mass action (reversible)

>Reaction 2

S + R = RS

Rate Law: Mass action (reversible)

>Reaction 3

C + RS = CRS

Rate Law: Mass action (reversible)

>Global quantities

Name	Simulation Type	Unit	Initial Value	Expression (M)
T	fixed	s	30	
h	assignment		0	If(Time lt Values[T],0,1)

>Parameter Overview

>Kinetic Parameters

reaction	
k1*	1.2 × 10^{6} M^{-1} s^{-1}
k2*	0.0014 s^{-1}
reaction_1	
k1**	2 × 10^{7} M^{-1} s^{-1}
k2**	6.8 × 10^{-5} s^{-1}
reaction_2	
k1**	2× 10^{7} M^{-1} s^{-1}
k2**	6.8 × 10^{-5} s^{-1}
reaction_3	
k1*	1.2 × 10^{6} M^{-1} s^{-1}
k2*	0.0014 s^{-1}

*Data are presented according to Safenkova et al. [5]

**Data are presented according to Chivers et al. [6]

>Mathematical

>Differential equations

$$\frac{d([R]\cdot V_{compartment})}{dt} = V_{compartment}\cdot(-(\text{"(reaction).k1"})\cdot[C]\cdot[R] + \text{"(reaction).k2"}\cdot[CR])$$

$$\frac{d([C]\cdot V_{compartment})}{dt} = V_{compartment}\cdot(-(\text{"(reaction).k1"})\cdot[C]\cdot[R] + \text{"(reaction).k2"}\cdot[CR])$$

$$\frac{d([CRS]\cdot V_{compartment})}{dt} = V_{compartment}\cdot((\text{"(reaction\_1).k1"}\cdot[CR]\cdot[S] - \text{"(reaction\_1).k2"}\cdot[CRS] + \text{"(reaction\_3).k1"}\cdot[RS]\cdot[C] - \text{"(reaction\_3).k2"}\cdot[CRS])\cdot h)$$

$$\frac{d([RS]\cdot V_{compartment})}{dt} = V_{compartment}\cdot((\text{"(reaction\_2).k1"}\cdot[R]\cdot[S] - \text{"(reaction\_2).k2"}\cdot[RS] - \text{"(reaction\_3).k1"}\cdot[RS]\cdot[C] + \text{"(reaction\_3).k2"}\cdot[CRS])\cdot h)$$

$$\frac{d([S]\cdot V_{compartment})}{dt} = V_{compartment}\cdot((-(\text{"(reaction\_1).k1"})\cdot[CR]\cdot[S] + \text{"(reaction\_1).k2"}\cdot[CRS] - \text{"(reaction\_2).k1"}\cdot[R]\cdot[S] + \text{"(reaction\_2).k2"}\cdot[RS])\cdot h)$$

$$\frac{d([CR]\cdot V_{compartment})}{dt} = V_{compartment}\cdot(\text{"(reaction).k1"}\cdot[C]\cdot[R] - \text{"(reaction).k2"}\cdot[CR])$$

$$h = \begin{cases} \text{Time} < \text{t1}, & 0 \\ \text{else}, & 1 \end{cases}$$

>Tasks

>Time course

Duration (s) – 300, Interval size (s) – 100, Intervals – 3

>Parameter scan

Object – [R]_0

Intervals – 10, min – 5×10^{-9}, max – 5×10^{-7}

Section S4. Characterization of gold nanoparticles (GNPs) and their conjugates with anti-fluorescein antibodies (antiFAM)

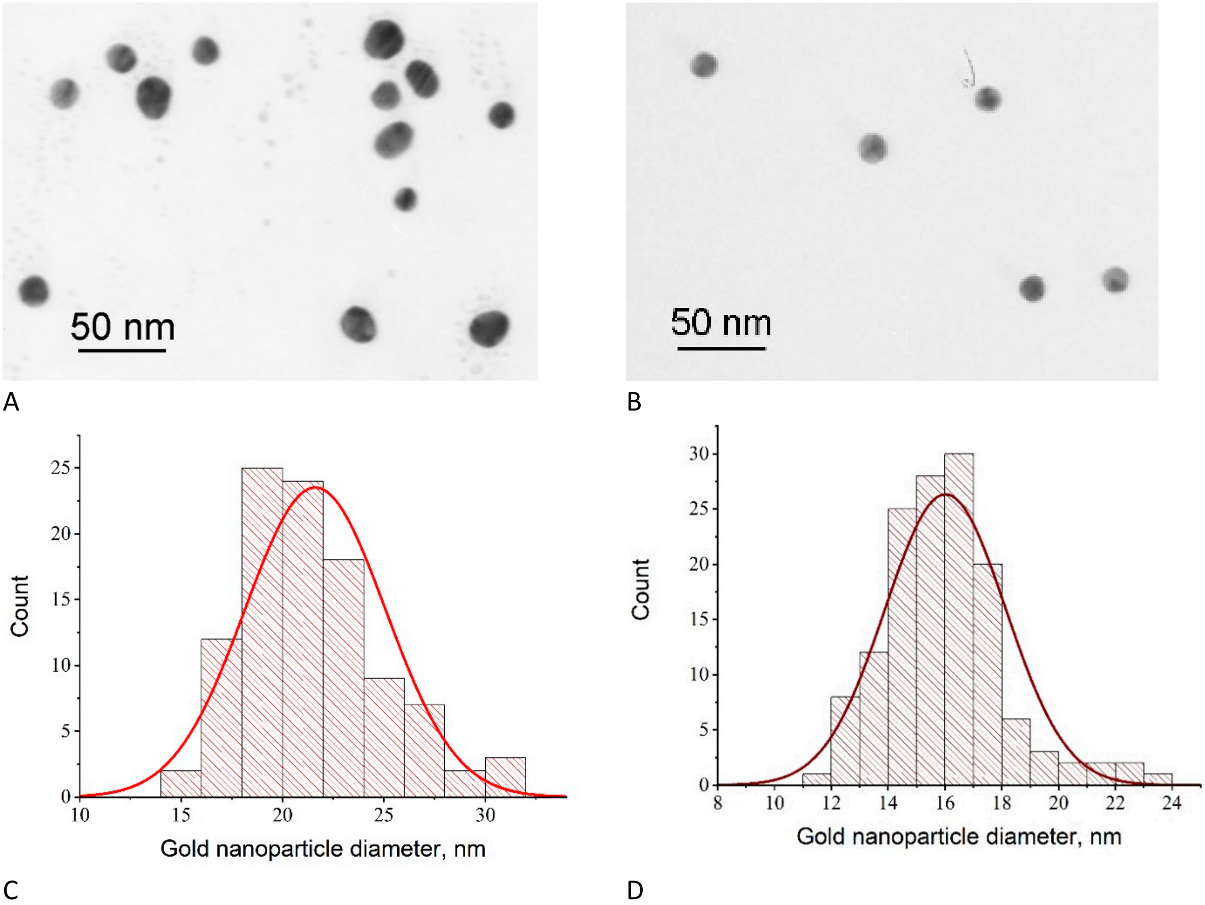

Figure S2. Characterization of the synthesized GNPs by TEM. Microphotographs of GNP_{21} (**A**) and GNP_{16} (**B**); diameter distributions and fitting by Gauss approximation according OriginPro 2021 software (Origin Lab, Northampton, MA, USA) for GNP_{21} (21.6 ± 3.5 nm, n = 102) (**C**) and GNP_{16} (16.0 ± 2.1 nm, n = 140) (**D**).

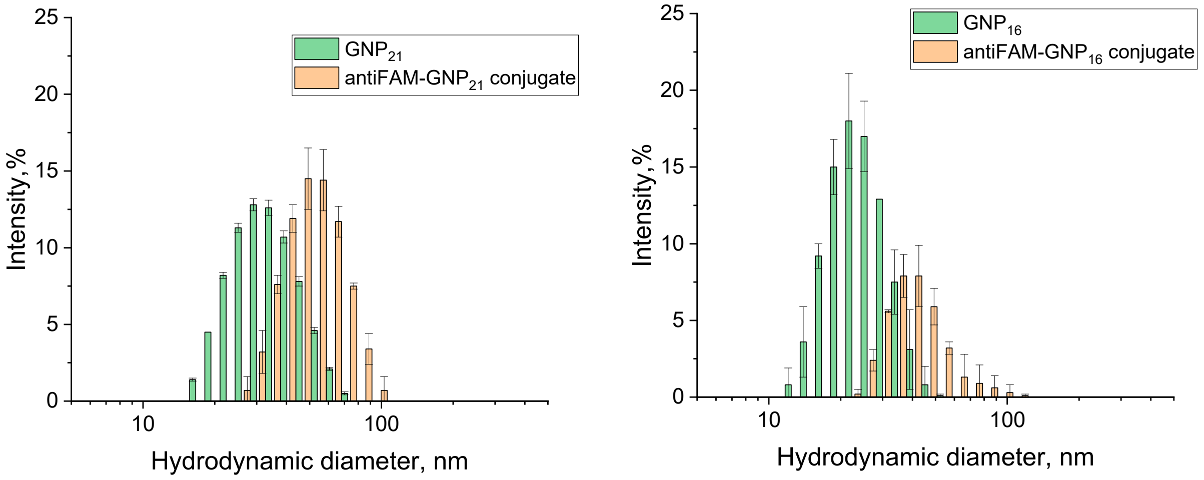

A B

Figure S3. Distribution of hydrodynamic diameters (D_H) of GNPs and antiFAM-GNP conjugates. (**A**) GNP_{21} (D_H = 32.1 ± 10.4 nm, polydispersity (Pd) = 32.4%) and antiFAM-GNP_{21} conjugate (D_H = 56.6 ± 14.2 nm, polydispersity (Pd) = 25.0%). (**B**) GNP_{16} (D_H = 23.5 ± 7.2 nm, polydispersity (Pd) = 30.5%) and antiFAM-GNP_{16} conjugate (D_H = 42.5 ± 8.4 nm, polydispersity (Pd) = 19.9%).

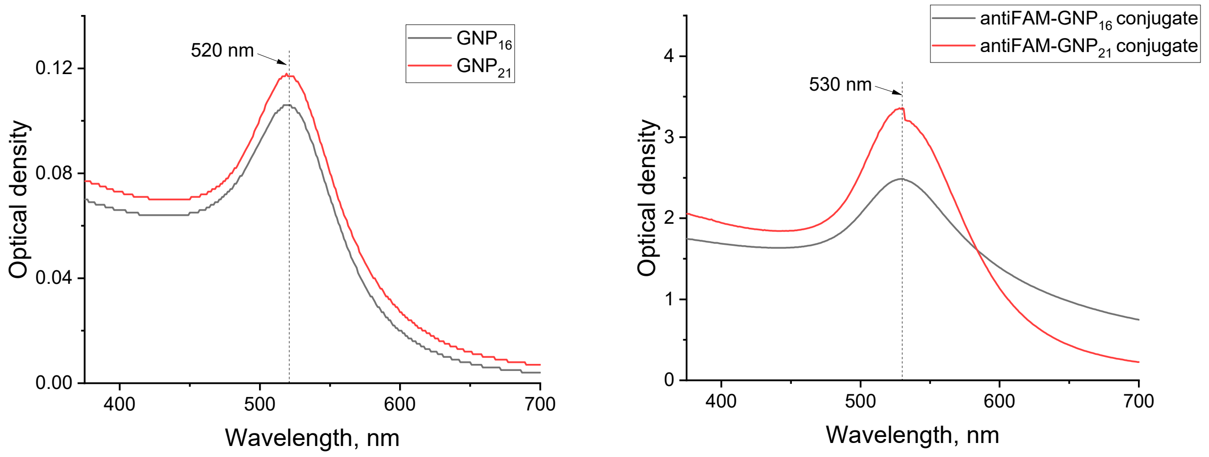

A B

Figure S4. Spectra of GNPs and antiFAM-GNP conjugates (optical path length = 1 mm). (**A**) GNP_{21} (λ_{max} = 520 nm) and GNP_{16} (λ_{max} = 520 nm). (**B**) antiFAM-GNP_{21} conjugate (λ_{max} = 530 nm) and antiFAM-GNP_{16} conjugate (λ_{max} = 530 nm).

Table S2. Comparative characteristics of gold nanoparticles

GNP	GNP diameter (TEM data), nm	GNP in the reaction mixture, OD at A520, optical path length = 1 cm	GNP, particles per mL
GNP_{16}	16.0 ± 2.1	1.0	1.2×10^{12}
GNP_{21}	21.6 ± 3.5	1.2	4.9×10^{11}

Section S5. Characterization of FAM-dT10-Bio reporter binding to antiFAM sites at different incubation time

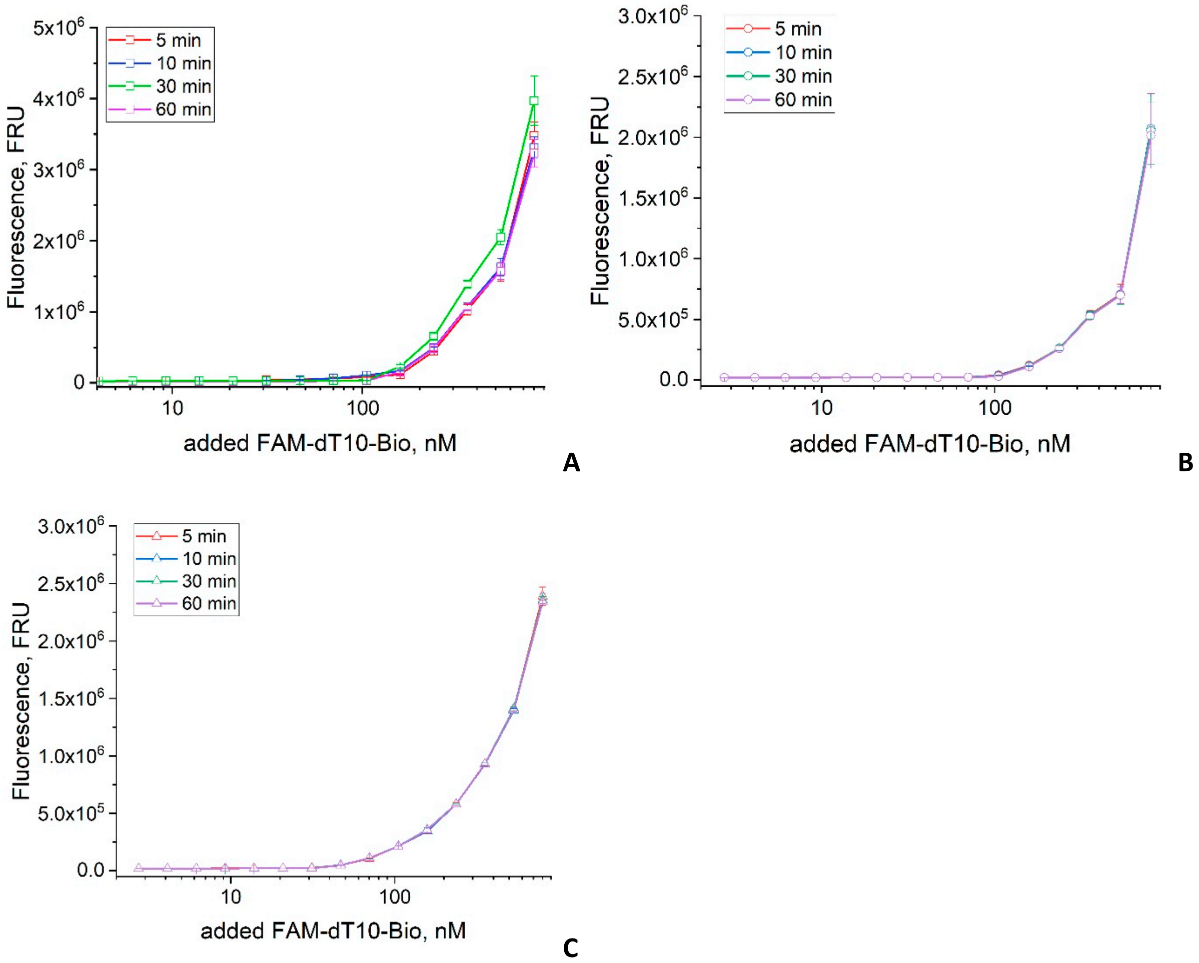

Figure S5. Characterization of FAM-dT10-Bio reporter binding to antiFAM sites at different incubation time. **(A)** Fluorescence responses for various reporter concentrations with addition of antibodies (10 µg/mL in the reaction mixture). **(B)** Fluorescence responses for various reporter concentrations with addition of antiFAM-GNP$_{16}$ conjugate (A_{520} = 1 in the reaction mixture). **(C)** Fluorescence responses for various reporter concentrations with addition of antiFAM-GNP$_{21}$ conjugate (A_{520} = 1 in the reaction mixture).

Section S6. Testing of reporter/LFT systems of different compositions with antiFAM-GNP conjugate immobilized on the membrane in the absence of cleaved reporters (negative experiment)

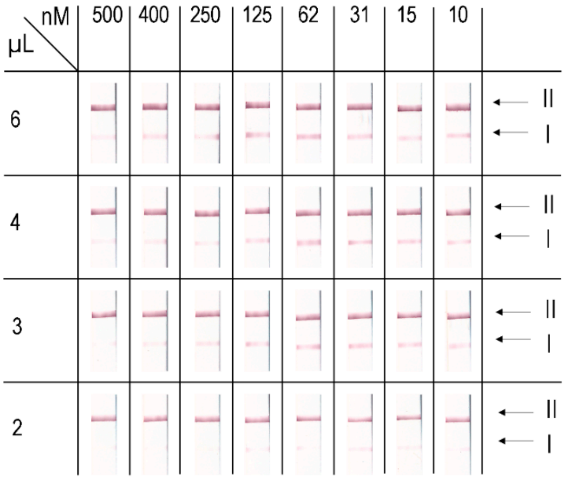

A

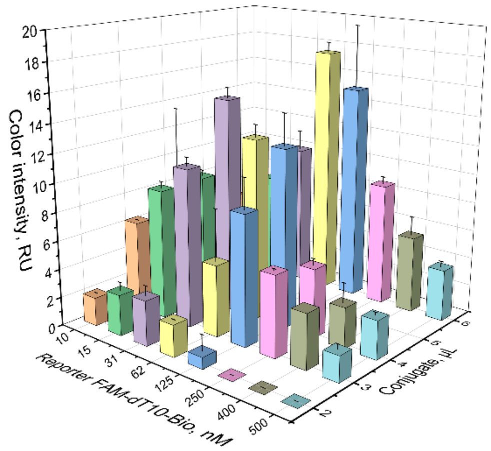

B

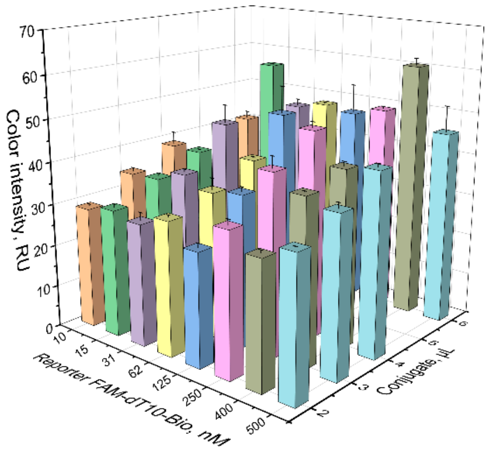

C

Figure S6. Testing of reporter/LFT systems of different compositions with **antiFAM-GNP$_{21}$ conjugate immobilized on the membrane** and **0.3 mg/mL streptavidin**. **(A)** Scans of LFT strips at different concentrations of reporter and antiFAM-GNP$_{21}$ conjugate (A_{520} = 3.0). I indicates the first (control) zone, II indicates the second (test) zone. **(B)** Dependences of color intensities in the first (control) zone on reporter and conjugate concentrations. **(C)** Dependence of color intensities in the second (test) zone on reporter and conjugate concentrations.

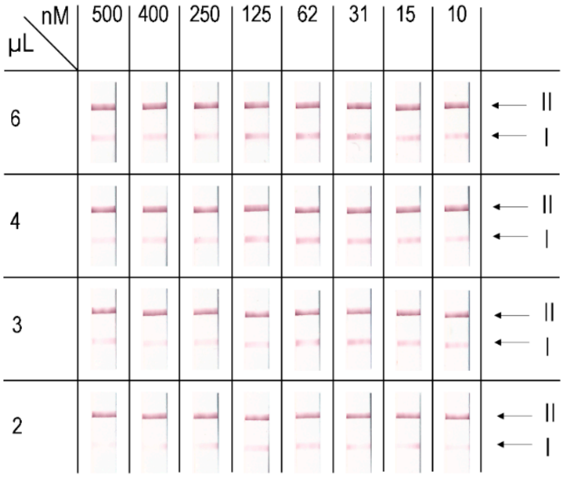

A

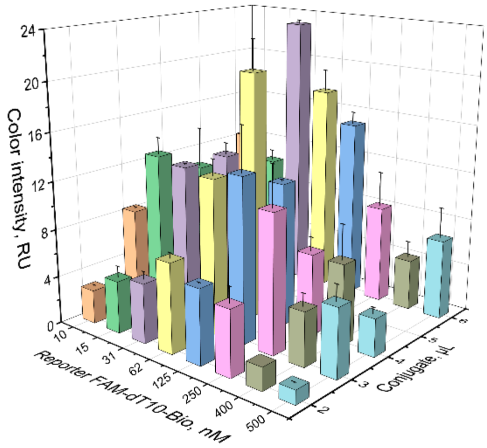

B

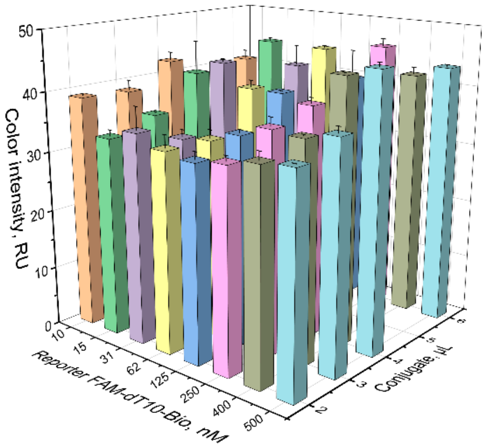

C

Figure S7. Testing of reporter/LFT systems of different compositions with **antiFAM-GNP$_{21}$ conjugate immobilized on the membrane** and **0.5 mg/mL streptavidin**. **(A)** Scans of LFT strips at different concentrations of reporter and antiFAM-GNP$_{21}$ conjugate (A_{520} = 3.0). I indicates the first (control) zone, II indicates the second (test) zone. **(B)** Dependences of color intensities in the first (control) zone on reporter and conjugate concentrations. **(C)** Dependence of color intensities in the second (test) zone on reporter and conjugate concentrations.

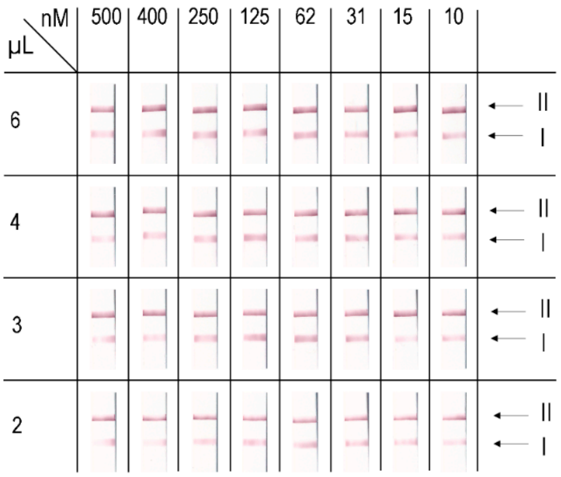

A

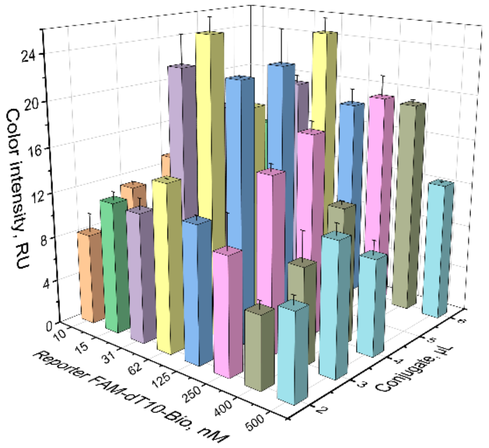

B

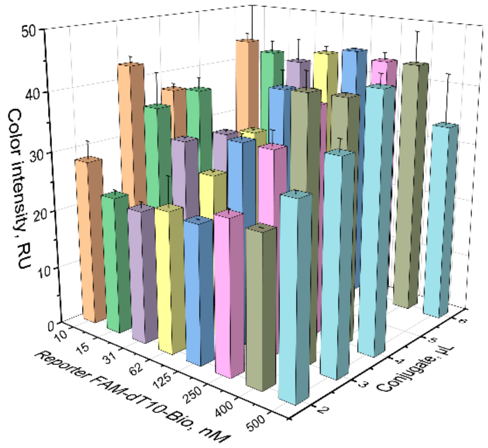

C

Figure S8. Testing of reporter/LFT systems of different compositions with **antiFAM-GNP$_{21}$ conjugate immobilized on the membrane** and **0.7 mg/mL streptavidin**. **(A)** Scans of LFT strips at different concentrations of reporter and antiFAM-GNP$_{21}$ conjugate (A_{520} = 3.0). I indicates the first (control) zone, II indicates the second (test) zone. **(B)** Dependences of color intensities in the first (control) zone on reporter and conjugate concentrations. **(C)** Dependence of color intensities in the second (test) zone on reporter and conjugate concentrations.

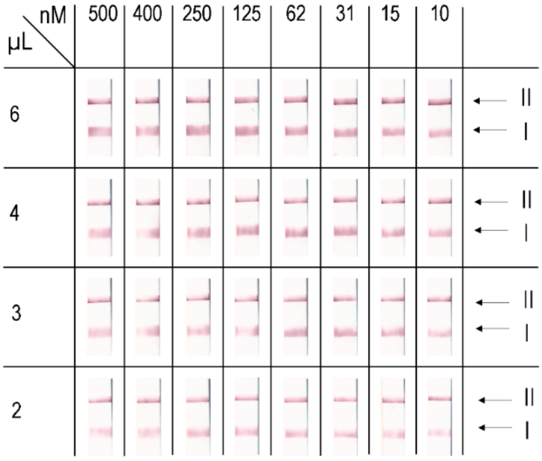

A

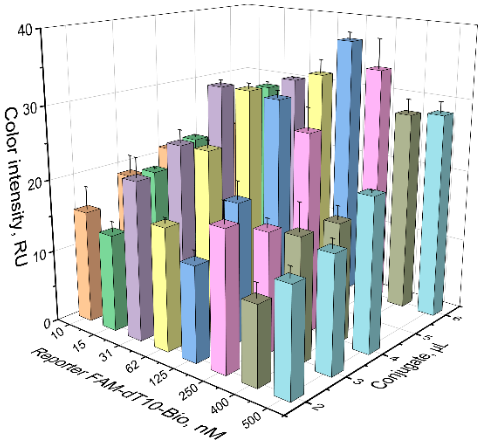

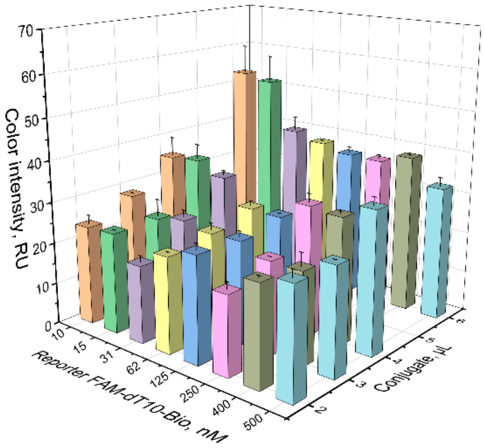

B C

Figure S9. Testing of reporter/LFT systems of different compositions with **antiFAM-GNP$_{21}$ conjugate immobilized on the membrane** and **1.0 mg/mL streptavidin**. **(A)** Scans of LFT strips at different concentrations of reporter and antiFAM-GNP$_{21}$ conjugate (A_{520} = 3.0). I indicates the first (control) zone, II indicates the second (test) zone. **(B)** Dependences of color intensities in the first (control) zone on reporter and conjugate concentrations. **(C)** Dependence of color intensities in the second (test) zone on reporter and conjugate concentrations.

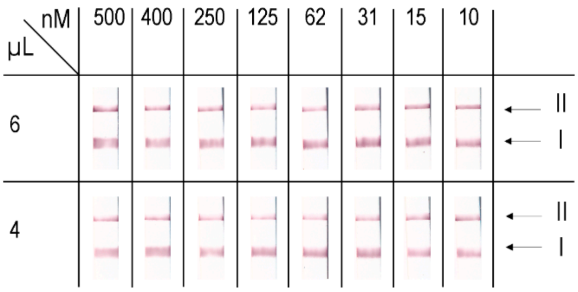

A

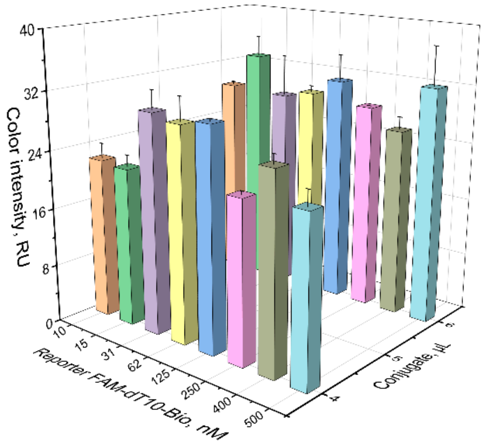

B

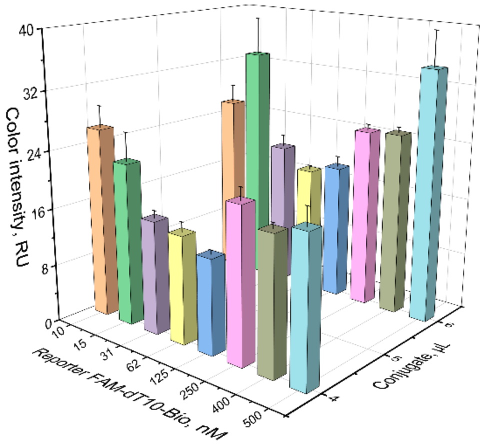

C

Figure S10. Testing of reporter/LFT systems of different compositions with **antiFAM-GNP$_{21}$ conjugate immobilized on the membrane** and **1.5 mg/mL streptavidin**. **(A)** Scans of LFT strips at different concentrations of reporter and antiFAM-GNP$_{21}$ conjugate (A_{520} = 3.0). I indicates the first (control) zone, II indicates the second (test) zone. **(B)** Dependences of color intensities in the first (control) zone on reporter and conjugate concentrations. **(C)** Dependence of color intensities in the second (test) zone on reporter and conjugate concentrations.

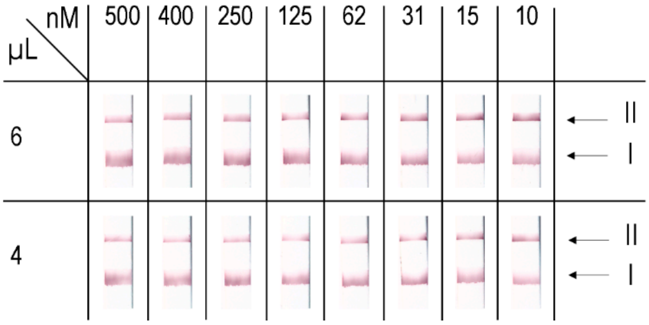

A

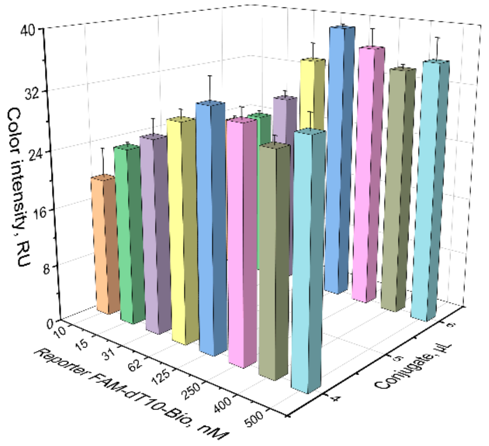

B

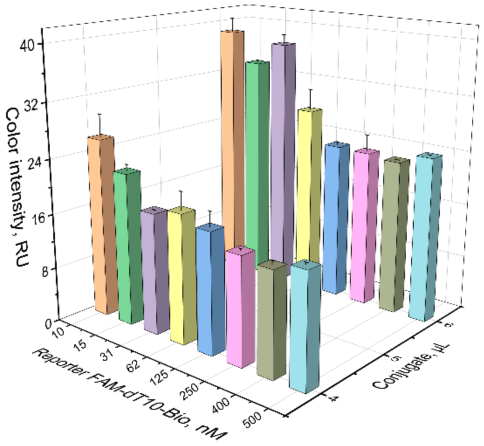

C

Figure S11. Testing of reporter/LFT systems of different compositions with **antiFAM-GNP$_{21}$ conjugate immobilized on the membrane** and **2.0 mg/mL streptavidin**. **(A)** Scans of LFT strips at different concentrations of reporter and antiFAM-GNP$_{21}$ conjugate (A_{520} = 3.0). I indicates the first (control) zone, II indicates the second (test) zone. **(B)** Dependences of color intensities in the first (control) zone on reporter and conjugate concentrations. **(C)** Dependence of color intensities in the second (test) zone on reporter and conjugate concentrations.

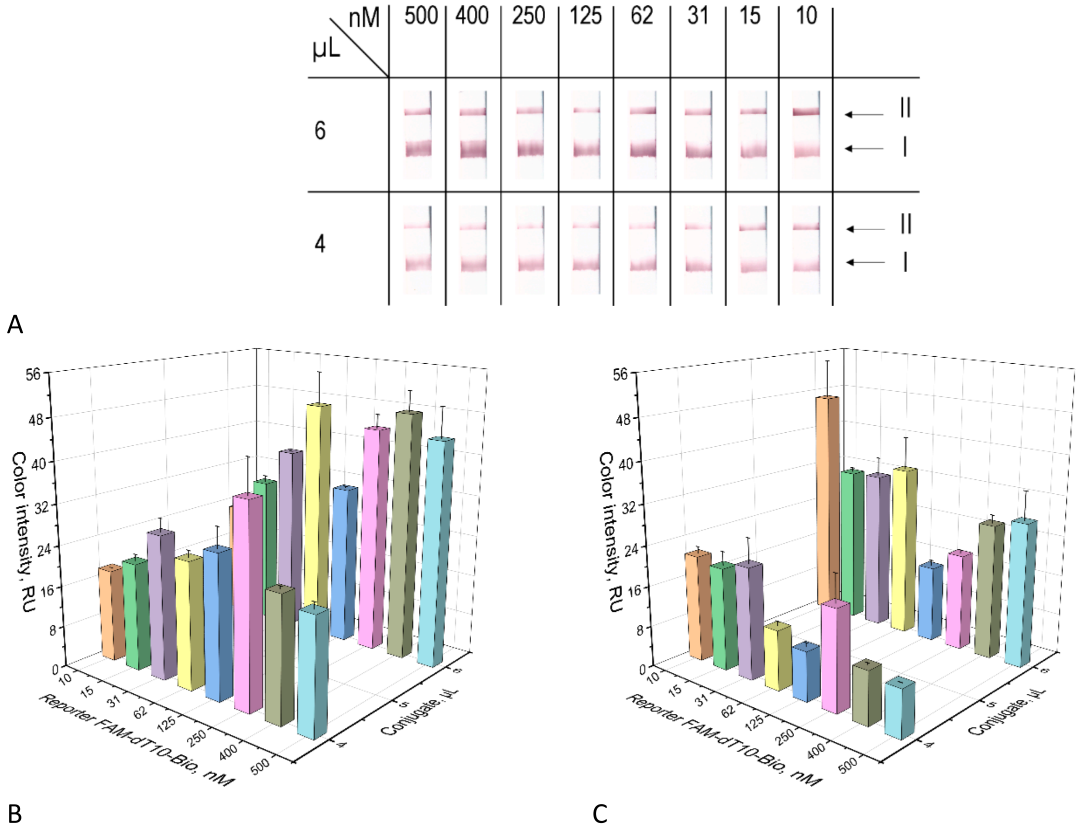

Figure S12. Testing of reporter/LFT systems of different compositions with **antiFAM-GNP$_{21}$ conjugate immobilized on the membrane** and **4.0 mg/mL streptavidin**. **(A)** Scans of LFT strips at different concentrations of reporter and antiFAM-GNP$_{21}$ conjugate (A_{520} = 3.0). I indicates the first (control) zone, II indicates the second (test) zone. **(B)** Dependences of color intensities in the first (control) zone on reporter and conjugate concentrations. **(C)** Dependence of color intensities in the second (test) zone on reporter and conjugate concentrations.

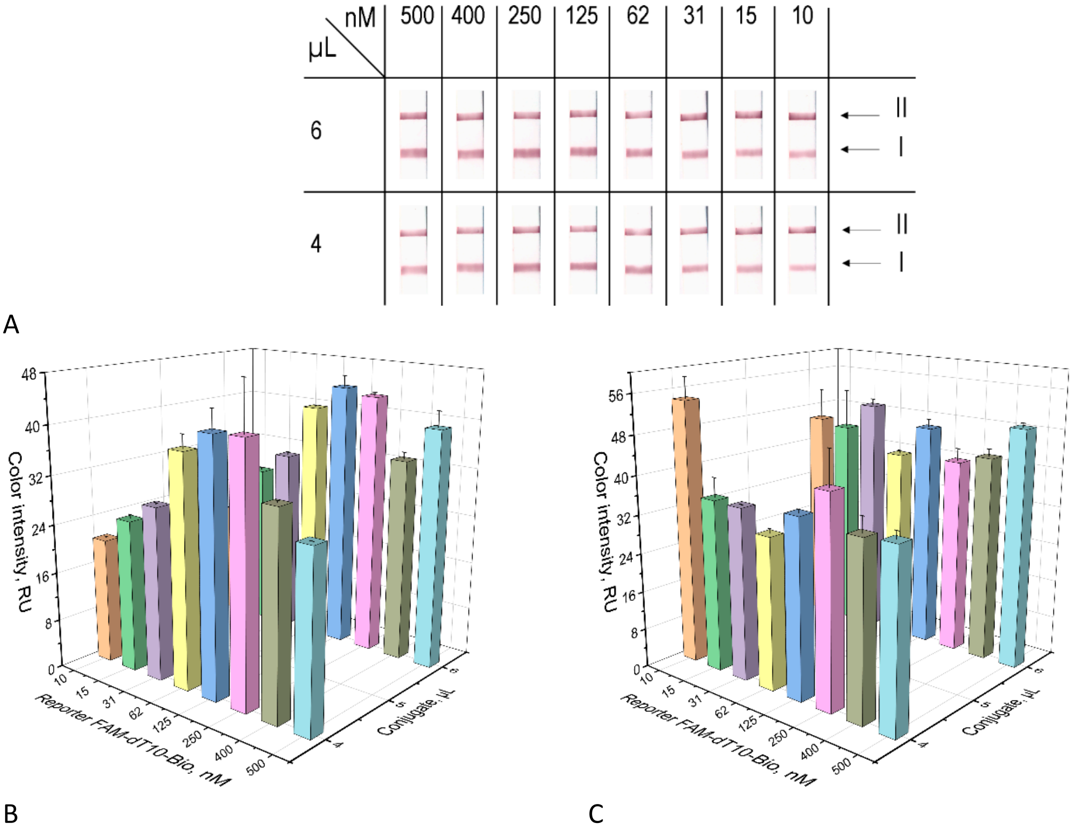

Figure S13. Testing of reporter/LFT systems of different compositions with **antiFAM-GNP$_{16}$ conjugate immobilized on the membrane** and **1.0 mg/mL streptavidin**. **(A)** Scans of LFT strips at different concentrations of reporter and antiFAM-GNP$_{16}$ conjugate (A_{520} = 3.0). I indicates the first (control) zone, II indicates the second (test) zone. **(B)** Dependences of color intensities in the first (control) zone on reporter and conjugate concentrations. **(C)** Dependence of color intensities in the second (test) zone on reporter and conjugate concentrations.

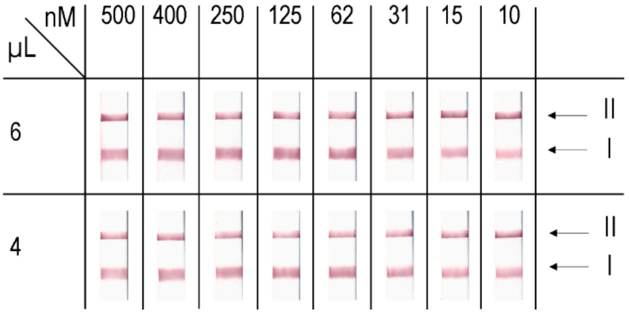

A

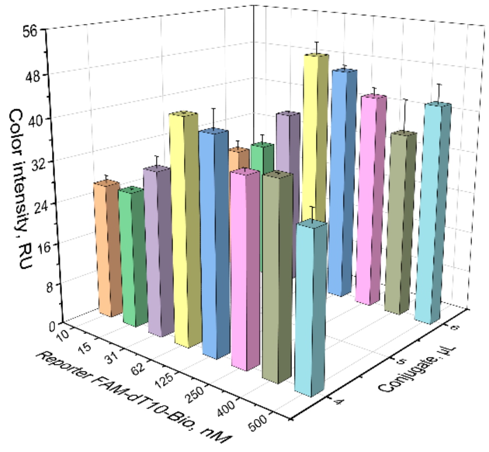

B

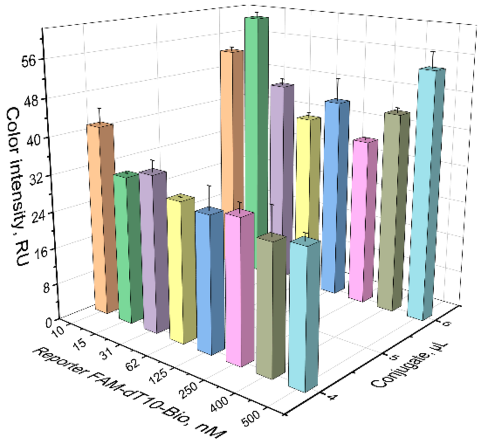

C

Figure S14. Testing of reporter/LFT systems of different compositions with **antiFAM-GNP$_{16}$ conjugate immobilized on the membrane** and **1.5 mg/mL streptavidin**. **(A)** Scans of LFT strips at different concentrations of reporter and antiFAM-GNP$_{16}$ conjugate (A_{520} = 3.0). I indicates the first (control) zone, II indicates the second (test) zone. **(B)** Dependences of color intensities in the first (control) zone on reporter and conjugate concentrations. **(C)** Dependence of color intensities in the second (test) zone on reporter and conjugate concentrations.

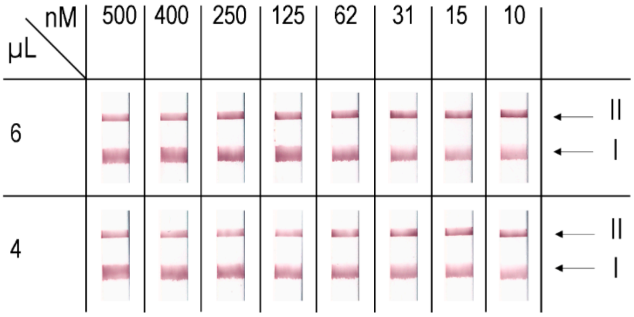

A

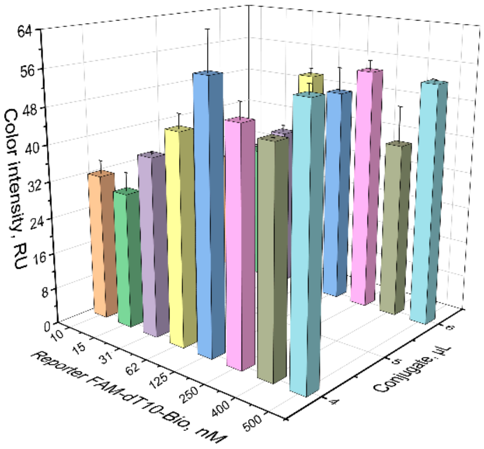

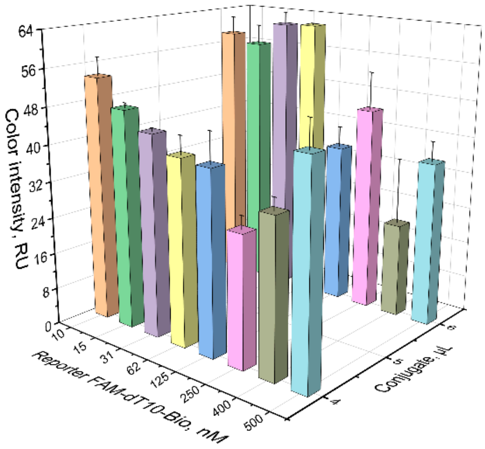

B C

Figure S15. Testing of reporter/LFT systems of different compositions with **antiFAM-GNP$_{16}$ conjugate immobilized on the membrane** and **2.0 mg/mL streptavidin**. **(A)** Scans of LFT strips at different concentrations of reporter and antiFAM-GNP$_{16}$ conjugate (A_{520} = 3.0). I indicates the first (control) zone, II indicates the second (test) zone. **(B)** Dependences of color intensities in the first (control) zone on reporter and conjugate concentrations. **(C)** Dependence of color intensities in the second (test) zone on reporter and conjugate concentrations.

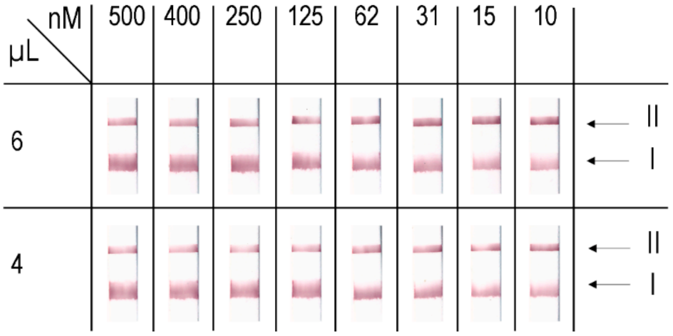

A

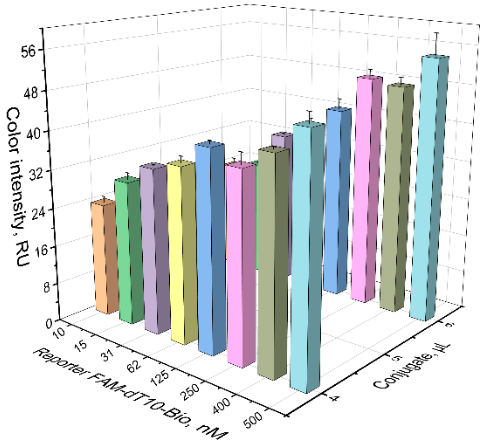

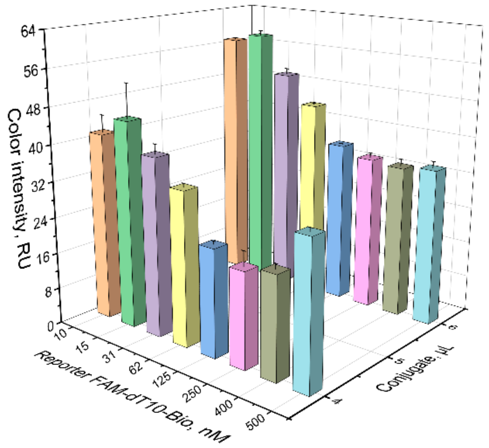

B C

Figure S16. Testing of reporter/LFT systems of different compositions with **antiFAM-GNP$_{16}$ conjugate immobilized on the membrane** and **4.0 mg/mL streptavidin**. **(A)** Scans of LFT strips at different concentrations of reporter and antiFAM-GNP$_{16}$ conjugate (A_{520} = 3.0). I indicates the first (control) zone, II indicates the second (test) zone. **(B)** Dependences of color intensities in the first (control) zone on reporter and conjugate concentrations. **(C)** Dependence of color intensities in the second (test) zone on reporter and conjugate concentrations.

Section S7. Testing of reporter/LFT systems of different compositions with antiFAM-GNP conjugate added directly to the reporter solution with 5 min pre-incubation in the absence of cleaved reporters (negative experiment)

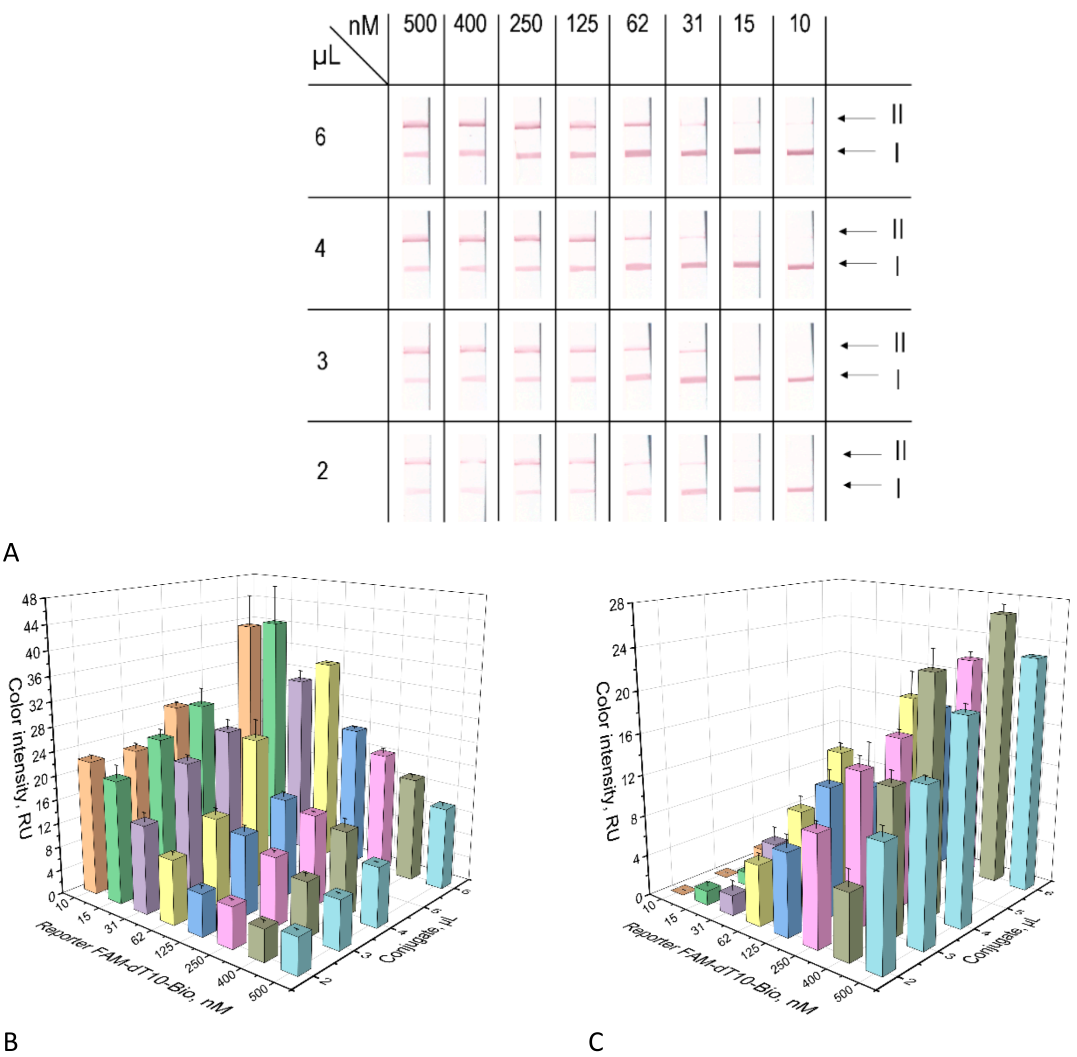

Figure S17. Testing of reporter/LFT systems of different compositions with **antiFAM-GNP$_{21}$ conjugate added directly to the reporter solution with 5 min pre-incubation** and **0.3 mg/mL streptavidin**. **(A)** Scans of LFT strips at different concentrations of reporter and antiFAM-GNP$_{21}$ conjugate (A_{520} = 3.0). I indicates the first (control) zone, II indicates the second (test) zone. **(B)** Dependences of color intensities in the first (control) zone on reporter and conjugate concentrations. **(C)** Dependence of color intensities in the second (test) zone on reporter and conjugate concentrations.

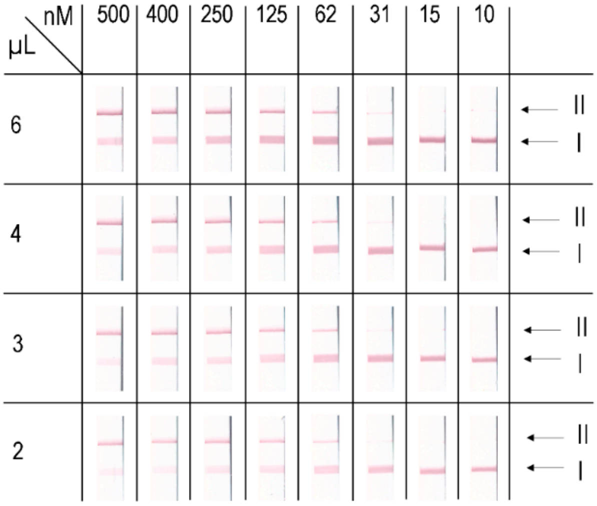

A

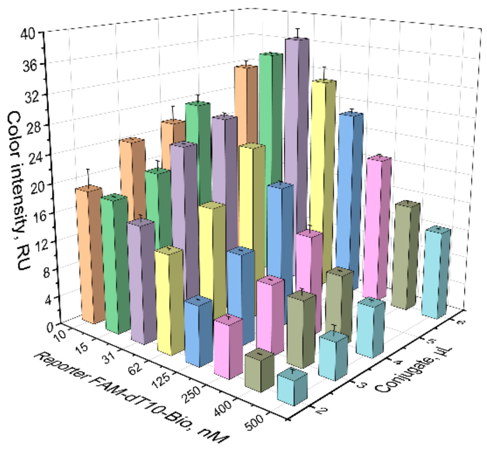

B

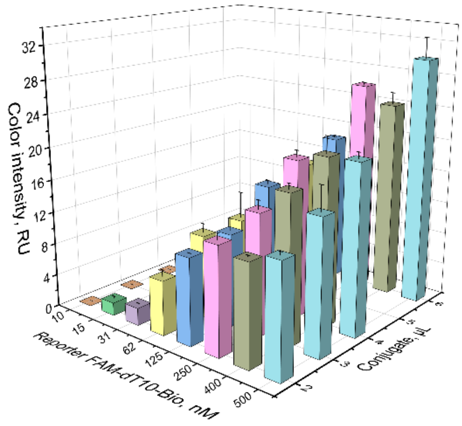

C

Figure S18. Testing of reporter/LFT systems of different compositions with **antiFAM-GNP$_{21}$ conjugate added directly to the reporter solution with 5 min pre-incubation** and **0.5 mg/mL streptavidin**. **(A)** Scans of LFT strips at different concentrations of reporter and antiFAM-GNP$_{21}$ conjugate (A_{520} = 3.0). I indicates the first (control) zone, II indicates the second (test) zone. **(B)** Dependences of color intensities in the first (control) zone on reporter and conjugate concentrations. **(C)** Dependence of color intensities in the second (test) zone on reporter and conjugate concentrations.

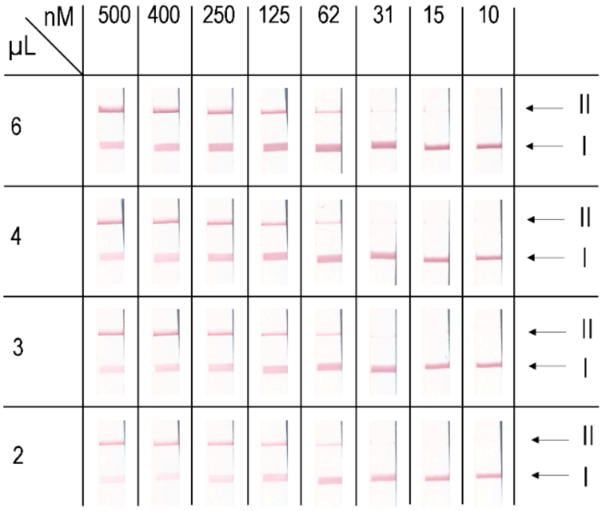

A

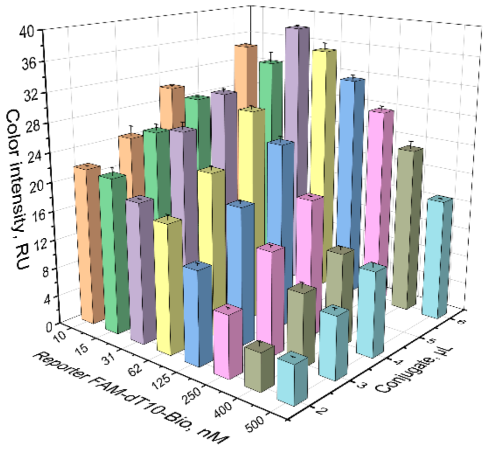

B

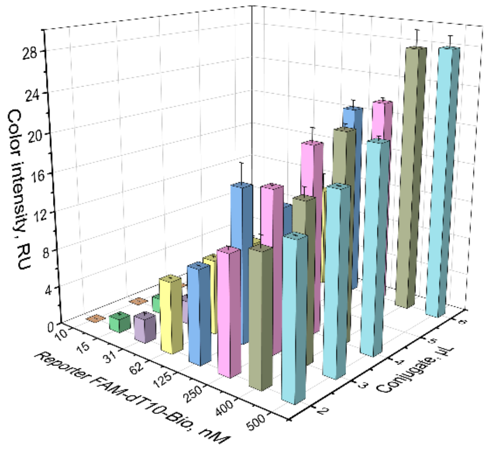

C

Figure S19. Testing of reporter/LFT systems of different compositions with **antiFAM-GNP$_{21}$ conjugate added directly to the reporter solution with 5 min pre-incubation** and **0.7 mg/mL streptavidin**. **(A)** Scans of LFT strips at different concentrations of reporter and antiFAM-GNP$_{21}$ conjugate (A_{520} = 3.0). I indicates the first (control) zone, II indicates the second (test) zone. **(B)** Dependences of color intensities in the first (control) zone on reporter and conjugate concentrations. **(C)** Dependence of color intensities in the second (test) zone on reporter and conjugate concentrations.

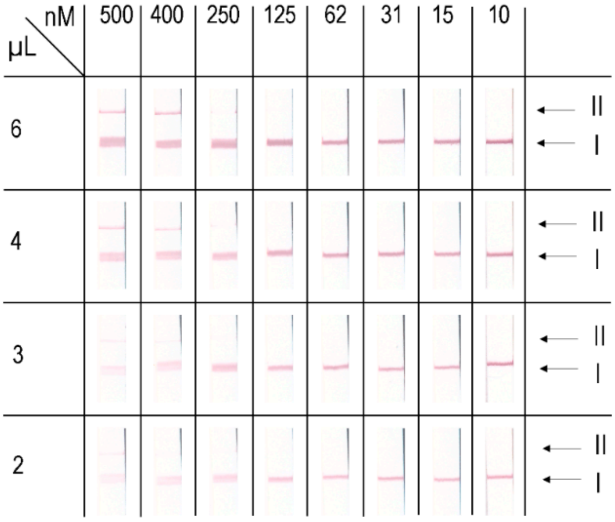

A

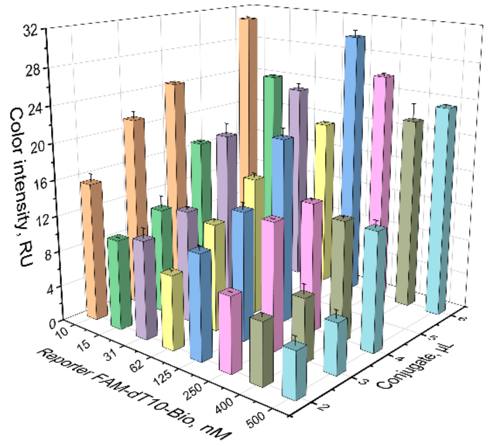

B

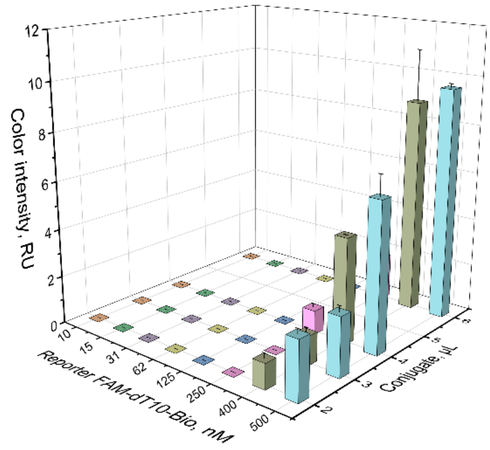

C

Figure S20. Testing of reporter/LFT systems of different compositions with **antiFAM-GNP$_{21}$ conjugate added directly to the reporter solution with 5 min pre-incubation** and **1.0 mg/mL streptavidin**. **(A)** Scans of LFT strips at different concentrations of reporter and antiFAM-GNP$_{21}$ conjugate (A_{520} = 3.0). I indicates the first (control) zone, II indicates the second (test) zone. **(B)** Dependences of color intensities in the first (control) zone on reporter and conjugate concentrations. **(C)** Dependence of color intensities in the second (test) zone on reporter and conjugate concentrations.

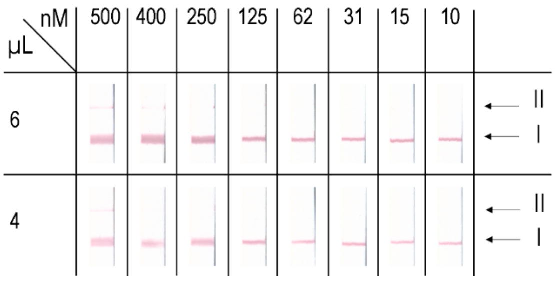

A

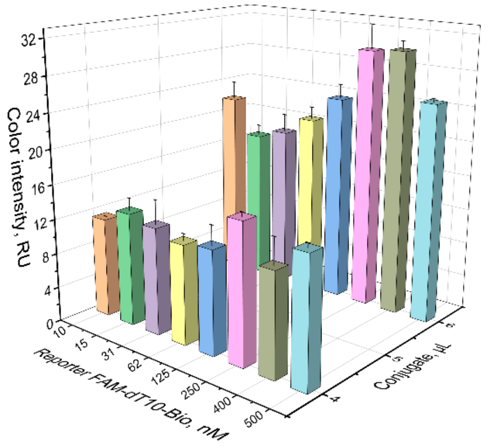

B

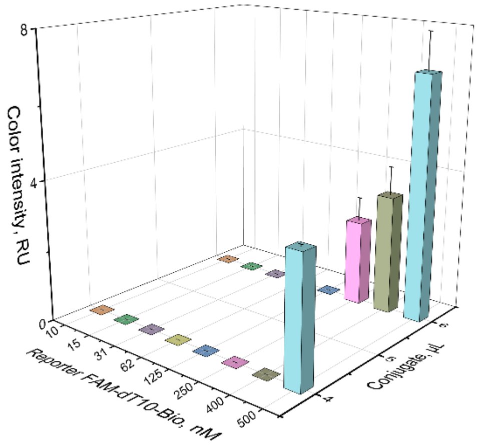

C

Figure S21. Testing of reporter/LFT systems of different compositions with **antiFAM-GNP$_{21}$ conjugate added directly to the reporter solution with 5 min pre-incubation** and **1.5 mg/mL streptavidin**. **(A)** Scans of LFT strips at different concentrations of reporter and antiFAM-GNP$_{21}$ conjugate (A_{520} = 3.0). I indicates the first (control) zone, II indicates the second (test) zone. **(B)** Dependences of color intensities in the first (control) zone on reporter and conjugate concentrations. **(C)** Dependence of color intensities in the second (test) zone on reporter and conjugate concentrations.

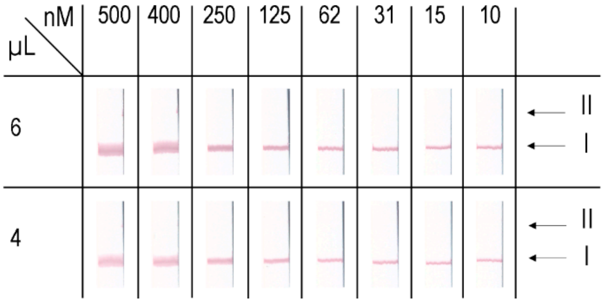

A

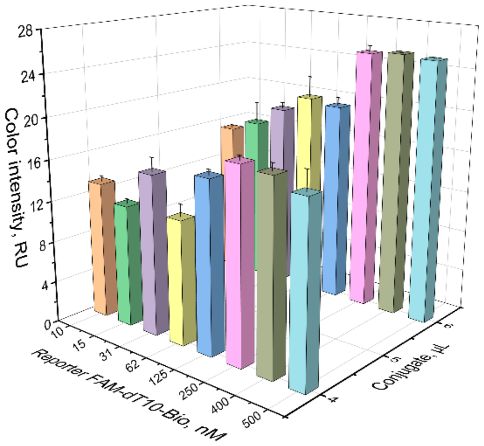

B

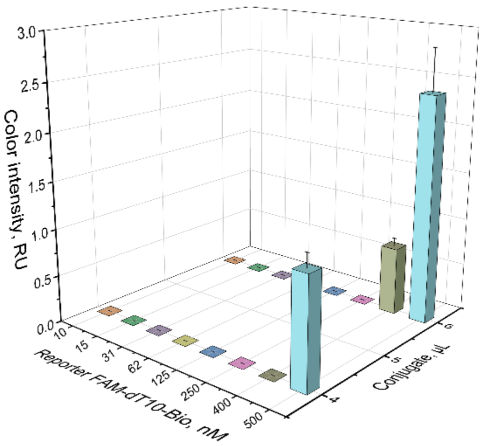

C

Figure S22. Testing of reporter/LFT systems of different compositions with **antiFAM-GNP$_{21}$ conjugate added directly to the reporter solution with 5 min pre-incubation** and **2.0 mg/mL streptavidin**. **(A)** Scans of LFT strips at different concentrations of reporter and antiFAM-GNP$_{21}$ conjugate (A_{520} = 3.0). I indicates the first (control) zone, II indicates the second (test) zone. **(B)** Dependences of color intensities in the first (control) zone on reporter and conjugate concentrations. **(C)** Dependence of color intensities in the second (test) zone on reporter and conjugate concentrations.

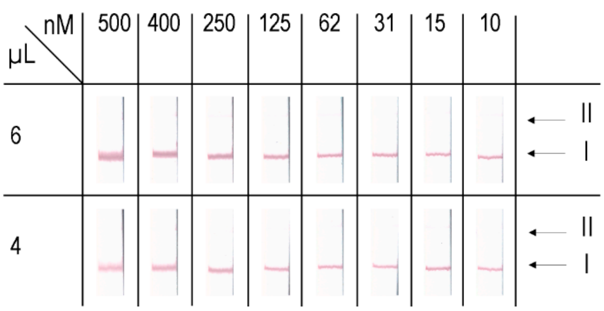

A

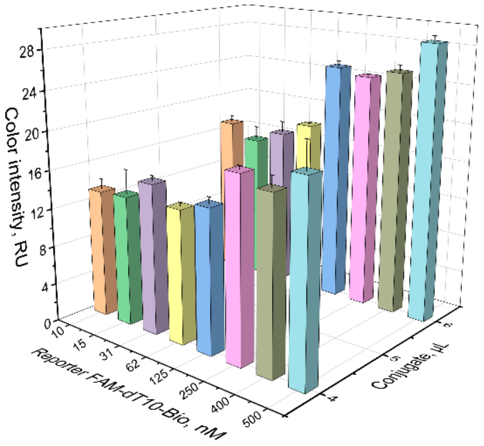

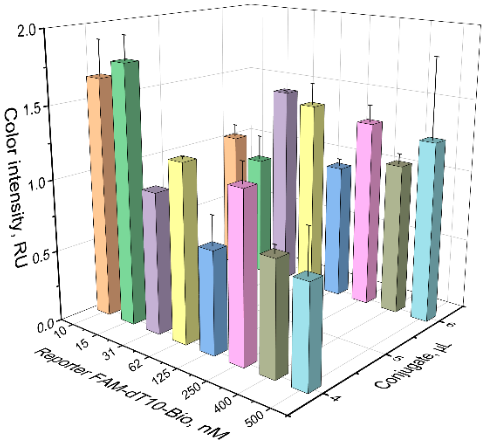

B C

Figure S23. Testing of reporter/LFT systems of different compositions with **antiFAM-GNP$_{21}$ conjugate added directly to the reporter solution with 5 min pre-incubation** and **4.0 mg/mL streptavidin**. **(A)** Scans of LFT strips at different concentrations of reporter and antiFAM-GNP$_{21}$ conjugate (A_{520} = 3.0). I indicates the first (control) zone, II indicates the second (test) zone. **(B)** Dependences of color intensities in the first (control) zone on reporter and conjugate concentrations. **(C)** Dependence of color intensities in the second (test) zone on reporter and conjugate concentrations.

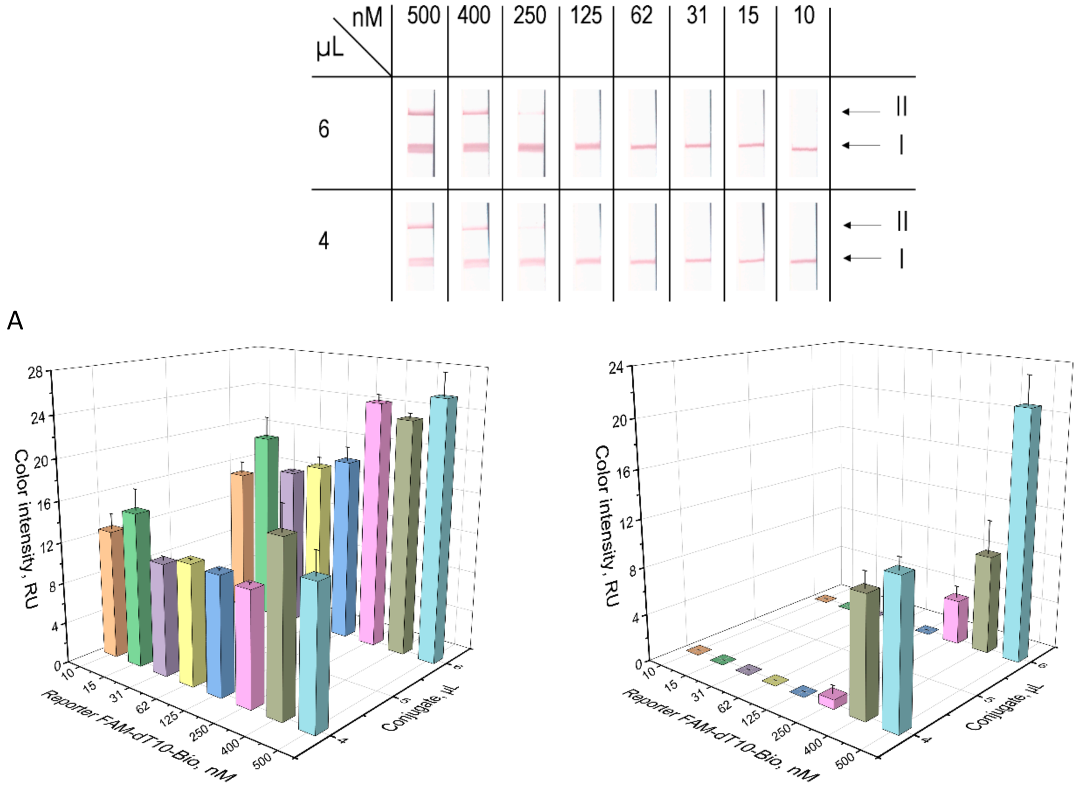

Figure S24. Testing of reporter/LFT systems of different compositions with **antiFAM-GNP$_{16}$ conjugate added directly to the reporter solution with 5 min pre-incubation** and **1.0 mg/mL streptavidin**. **(A)** Scans of LFT strips at different concentrations of reporter and antiFAM-GNP$_{16}$ conjugate (A_{520} = 3.0). I indicates the first (control) zone, II indicates the second (test) zone. **(B)** Dependences of color intensities in the first (control) zone on reporter and conjugate concentrations. **(C)** Dependence of color intensities in the second (test) zone on reporter and conjugate concentrations.

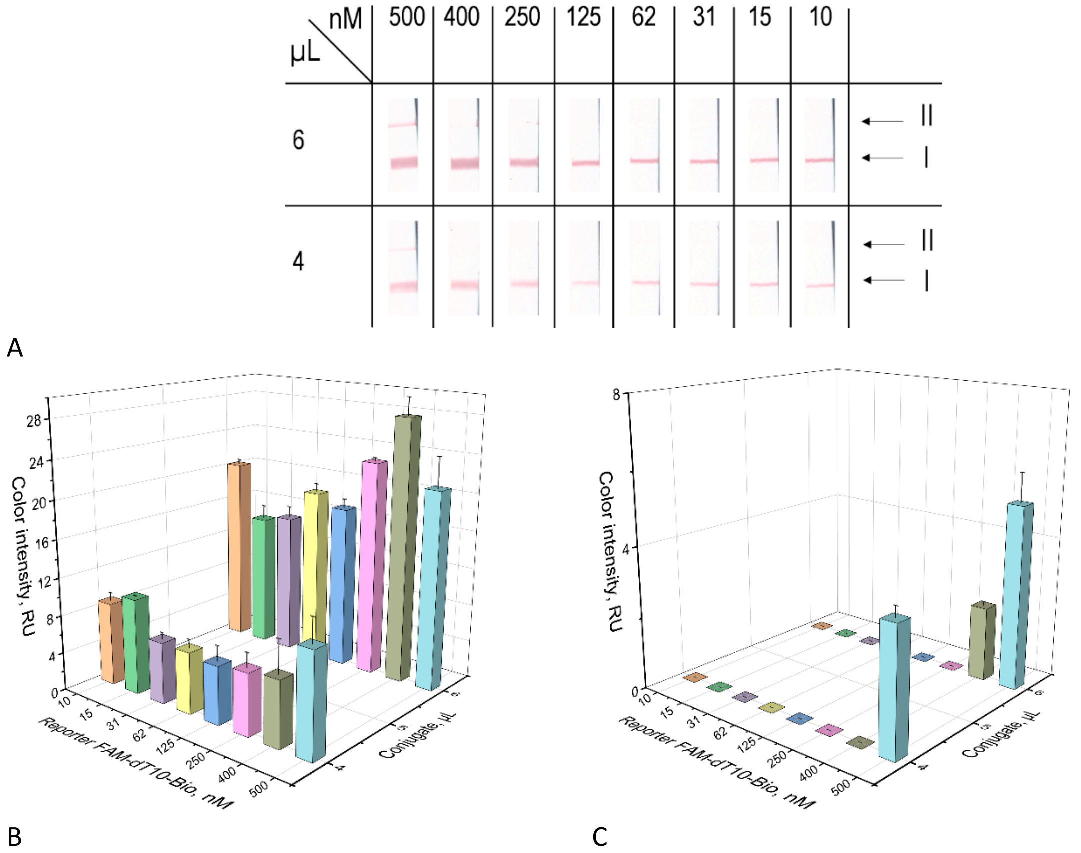

Figure S25. Testing of reporter/LFT systems of different compositions with **antiFAM-GNP$_{16}$ conjugate added directly to the reporter solution with 5 min pre-incubation** and **1.5 mg/mL streptavidin**. **(A)** Scans of LFT strips at different concentrations of reporter and antiFAM-GNP$_{16}$ conjugate (A_{520} = 3.0). I indicates the first (control) zone, II indicates the second (test) zone. **(B)** Dependences of color intensities in the first (control) zone on reporter and conjugate concentrations. **(C)** Dependence of color intensities in the second (test) zone on reporter and conjugate concentrations.

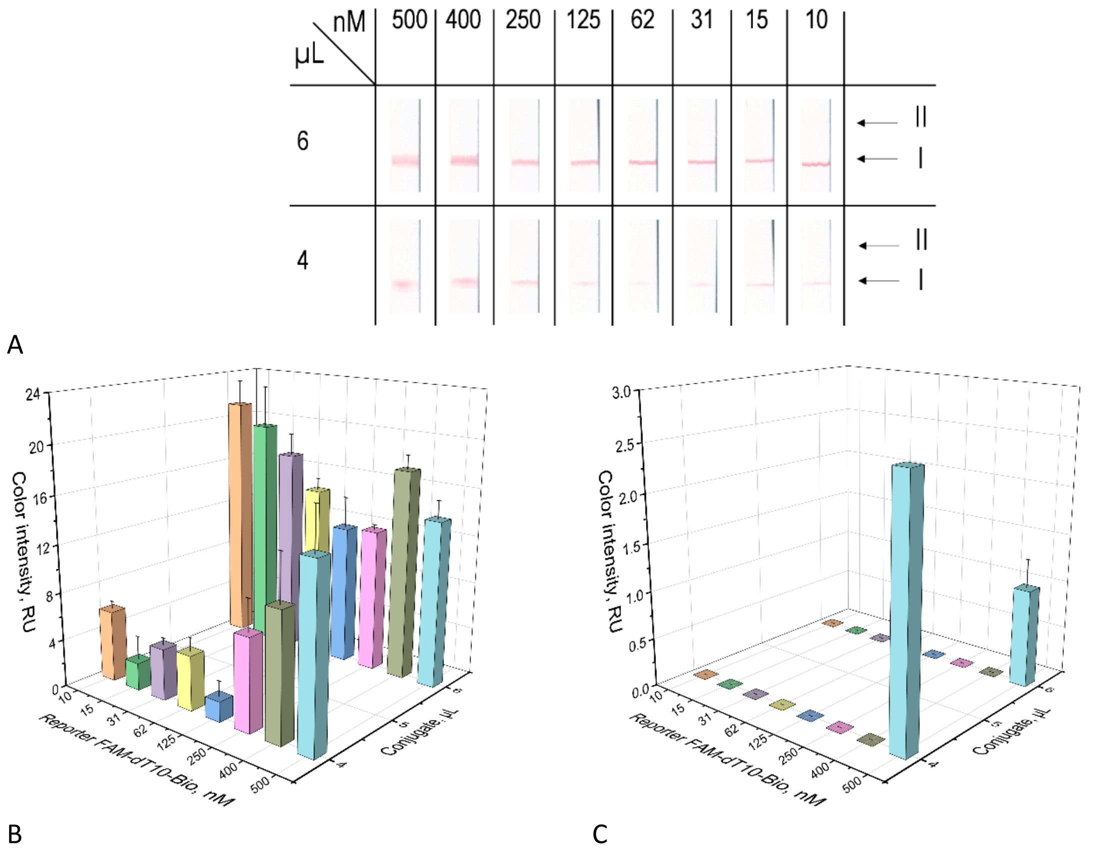

Figure S26. Testing of reporter/LFT systems of different compositions with **antiFAM-GNP$_{16}$ conjugate added directly to the reporter solution with 5 min pre-incubation** and **2.0 mg/mL streptavidin**. **(A)** Scans of LFT strips at different concentrations of reporter and antiFAM-GNP$_{16}$ conjugate (A_{520} = 3.0). I indicates the first (control) zone, II indicates the second (test) zone. **(B)** Dependences of color intensities in the first (control) zone on reporter and conjugate concentrations. **(C)** Dependence of color intensities in the second (test) zone on reporter and conjugate concentrations.

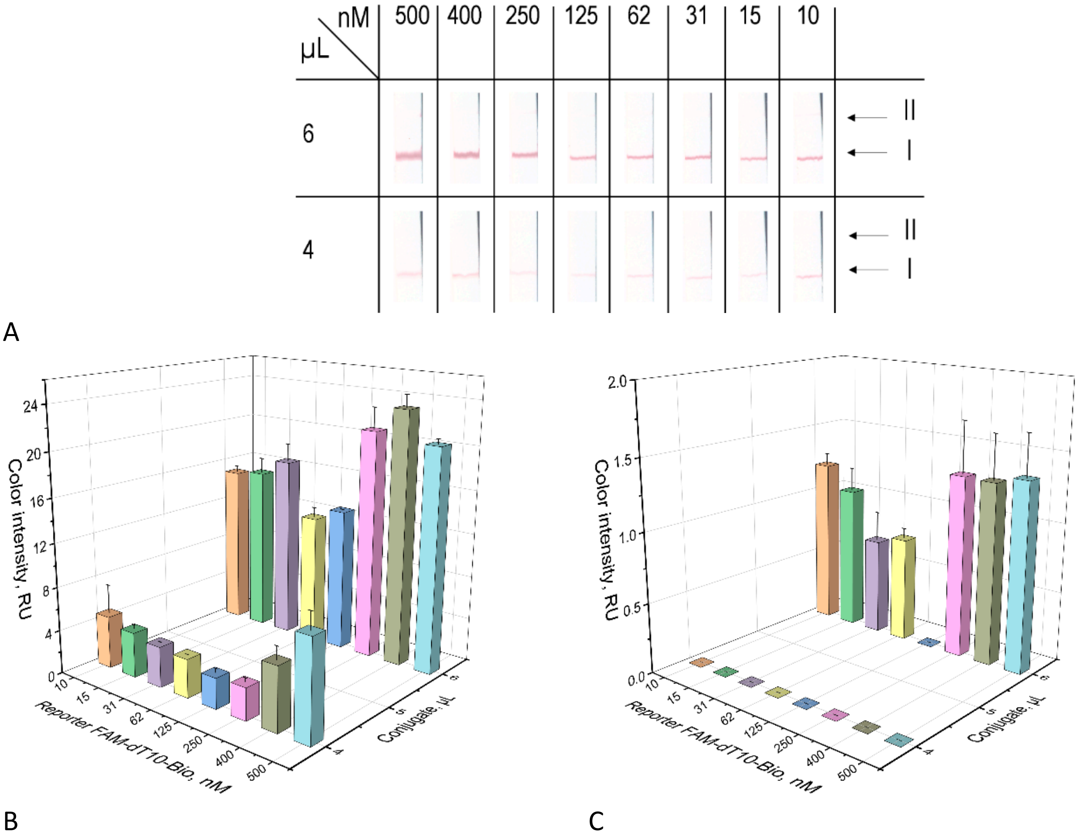

Figure S27. Testing of reporter/LFT systems of different compositions with **antiFAM-GNP$_{16}$ conjugate immobilized on the membrane** and **4.0 mg/mL streptavidin**. **(A)** Scans of LFT strips at different concentrations of reporter and antiFAM-GNP$_{16}$ conjugate (A_{520} = 3.0). I indicates the first (control) zone, II indicates the second (test) zone. **(B)** Dependences of color intensities in the first (control) zone on reporter and conjugate concentrations. **(C)** Dependence of color intensities in the second (test) zone on reporter and conjugate concentrations.

Section S8. Characterization of dsDNA target of *Erwinia amylovora* obtained by PCR

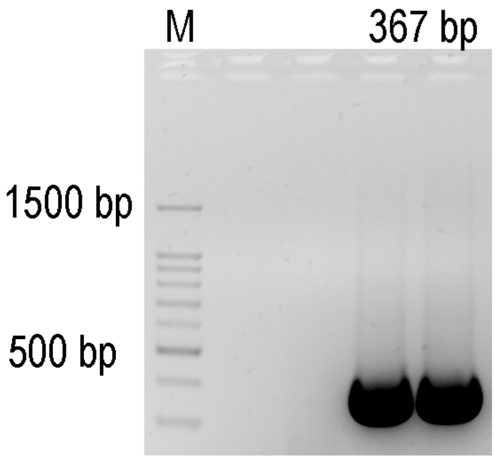

Figure S28. Visualization of PCR products (DNA fragment of *hisZ* gene with length of 367 bp) after electrophoresis in 2% agarose gel for the *E. amylovora*. M is the DNA marker.

Section S9. Detection of dsDNA-target in CRISPR/Cas12a with LFT detection

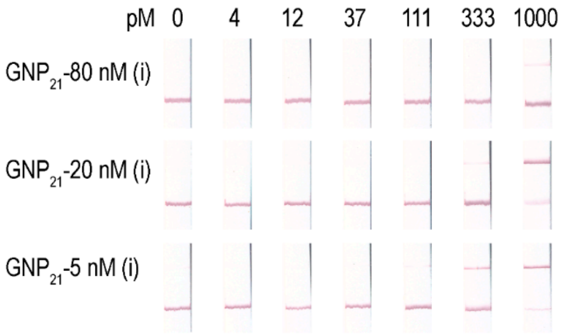

A

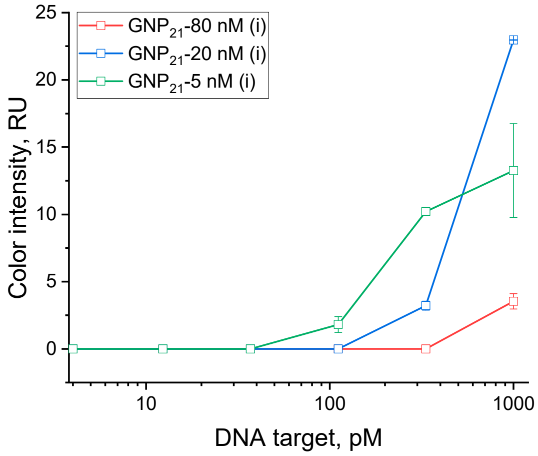

B

Figure S29. Detection of dsDNA-target (367 bp fragment corresponding to *hisZ* gene of *E. amylovora*) at various concentrations (4, 12, 37, 111, 333, 1000 pM) in CRISPR/Cas12a reactions. **(A)** Test strips (streptavidin 2 mg/mL, antiFAM-GNP$_{21}$ conjugate in solution A_{520} = 3.0, 6 µL) after CRISPR/Cas12a assay with FAM-dT10-Bio reporter (i) at different concentrations: 5, 20, 80 nM. **(B)** Concentration dependences of color intensities (test zone LFT) after CRISPR/Cas12a assay.

References

1. Ivanov, A. V.; Safenkova, I. V.; Drenova, N. V.; Zherdev, A. V.; Dzantiev, B. B., Comparison of Biosensing Methods Based on Different Isothermal Amplification Strategies: A Case Study with Erwinia amylovora. *Biosensors* **2022,** 12, (12), 1174.
2. Bühlmann, A.; Pothier, J. F.; Rezzonico, F.; Smits, T. H. M.; Andreou, M.; Boonham, N.; Duffy, B.; Frey, J. E., Erwinia amylovora loop-mediated isothermal amplification (LAMP) assay for rapid pathogen detection and on-site diagnosis of fire blight. *J Microbiol Meth* **2013,** 92, (3), 332-339.
3. Sotnikov, D. V.; Byzova, N.; Zherdev, A. V.; Dzantiev, B. B., Changes in antigen-binding ability of antibodies caused by immobilization on gold nanoparticles: A case study for monoclonal antibodies to fluorescein. *Biointer. Res. Appl. Chem* **2023,** 13, 550.
4. Sotnikov, D. V.; Byzova, N. A.; Zherdev, A. V.; Dzantiev, B. B., Ability of Antibodies Immobilized on Gold Nanoparticles to Bind Small Antigen Fluorescein. *Int J Mol Sci* **2023,** 24, (23), 16967.
5. Safenkova, I. V.; Ivanov, A. V.; Slutskaya, E. S.; Samokhvalov, A. V.; Zherdev, A. V.; Dzantiev, B. B., Key significance of DNA-target size in lateral flow assay coupled with recombinase polymerase amplification. *Anal. Chim. Acta* **2020,** 1102, 109-118.
6. Chivers, C. E.; Crozat, E.; Chu, C.; Moy, V. T.; Sherratt, D. J.; Howarth, M., A streptavidin variant with slower biotin dissociation and increased mechanostability. *Nat. Methods* **2010,** 7, (5), 391-393.
